# The salivary effector protein Sg2204 in the greenbug *Schizaphis graminum* suppresses wheat defence and is essential for enabling aphid feeding on host plants

**DOI:** 10.1111/pbi.13900

**Published:** 2022-08-19

**Authors:** Yong Zhang, Xiaobei Liu, Frédéric Francis, Haicui Xie, Jia Fan, Qian Wang, Huan Liu, Yu Sun, Julian Chen

**Affiliations:** ^1^ State Key Laboratory for Biology of Plant Diseases and Insect Pests Institute of Plant Protection, Chinese Academy of Agricultural Sciences Beijing China; ^2^ Functional and Evolutionary Entomology, Gembloux Agro‐Bio Tech University of Liège Gembloux Belgium; ^3^ College of Agronomy and Biotechnology Hebei Normal University of Science and Technology Qinhuangdao City China; ^4^ Department of Entomology College of Plant Protection China Agricultural University Beijing China

**Keywords:** *Schizaphis graminum*, salivary proteins, wheat, defence responses, nanocarrier‐mediated RNAi, aphid performance

## Abstract

Aphids secrete diverse repertoires of salivary effectors into host plant cells to promote infestation by modulating plant defence. The greenbug *Schizaphis graminum* is an important cereal aphid worldwide. However, the secreted effectors of *S. graminum* are still uncharacterized. Here, 76 salivary proteins were identified from the watery saliva of *S. graminum* using transcriptome and proteome analyses. Among them, a putative salivary effector Sg2204 was significantly up‐regulated during aphid feeding stages, and transient overexpression of Sg2204 in *Nicotiana benthamiana* inhibited cell death induced by BAX or INF1. Delivering Sg2204 into wheat via the type III secretion system of *Pseudomonas fluorescens* EtAnH suppressed pattern‐triggered immunity (PTI)‐associated callose deposition. The transcript levels of jasmonic acid (JA)‐ and salicylic acid (SA)‐associated defence genes of wheat were significantly down‐regulated, and the contents of both JA and SA were also significantly decreased after delivery of Sg2204 into wheat leaves. Additionally, feeding on wheat expressing Sg2204 significantly increased the weight and fecundity of *S. graminum* and promoted aphid phloem feeding. *Sg2204* was efficiently silenced via spray‐based application of the nanocarrier‐mediated transdermal dsRNA delivery system. Moreover, *Sg2204*‐silenced aphids induced a stronger wheat defence response and resulted in negative impacts on aphid feeding behaviour, survival and fecundity. Silencing of *Sg2204* homologues from four aphid species using nanocarrier‐delivered dsRNA also significantly reduced aphid performance on host plants. Thus, our study characterized the salivary effector Sg2204 of *S. graminum* involved in promoting host susceptibility by suppressing wheat defence, which can also be regarded as a promising RNAi target for aphid control.

## Introduction

Greenbug *Schizaphis graminum* is one of the most important and devastating cereal aphids in the world because it draws phloem sap and serves as a vector for transmitting viruses (Blackman and Eastop, 2000). In contrast to most other aphid species, *S. graminum* is a phytotoxic aphid, and its feeding can rapidly induce obvious leaf chlorosis in susceptible plants, resulting in the deterioration of plant quality and even plant death (Al‐Mousawi *et al*., 1983; Blackman and Eastop, 2000; Nicholson and Puterka, 2014; Zhang *et al*., 2019). A previous study showed that induced hydrogen peroxide accumulation is predicted to be involved in the induction of chlorosis symptoms by *S. graminum* feeding (Zhang *et al*., 2020). As global warming continues, the potential risk of *S. graminum* infestation will increase, especially in the Northern Hemisphere, which could increase global food insecurity and poverty by invading economically important crops (Aljaryian and Kumar, 2016).

Plants have evolved complicated defence systems against pathogens and insect herbivores (Schuman and Baldwin, 2016). However, during infection, pathogens produce an arsenal of effectors that are thought to be major determinants of pathogen virulence in host plants (Dou and Zhou, 2012; Jones and Dangl, 2006). Effectors secreted by pathogens enter host plant cells to suppress plant defence responses and promote plant susceptibility (Giraldo and Valent, 2013). Recent evidence suggests that aphids, similar to plant pathogens, present conserved molecules in their saliva similar to pathogen‐associated molecular patterns (PAMPs), which are perceived by pattern recognition receptors (PRRs) on the plant cell membrane and trigger PAMP‐triggered immunity (PTI). Aphids then secrete effectors that suppress this and other types of plant defences and promote effector‐triggered susceptibility (ETS). Nevertheless, effectors that are deployed to suppress host defences are recognized by plant resistance (R) proteins, activating a strong immune response called effector‐triggered immunity (ETI), typically including a hypersensitive response (Hogenhout and Bos, 2011; Kaloshian and Walling, 2005; Rodriguez and Bos, 2013; van Bel and Will, 2016).

During probing and feeding, aphid secreted saliva containing virulence proteins (effectors) into plant cytoplasm and modulate plant defence pathways (Mugford *et al*., 2016). Silencing of *C002*, which is specifically expressed in the salivary gland of aphids, leads to lethality in the pea aphid *Acyrthosiphon pisum* (Mutti *et al*., 2006, 2008). Transit overexpression of salivary effectors MpC002, Mp1/PIntO1 and PIntO2 of the green peach aphid *Myzus persicae* in tobacco *Nicotiana benthamiana* or *Arabidopsis* promotes aphid fecundity (Bos *et al*., 2010; Pitino and Hogenhout, 2013). The salivary effector Mp55 of *M. persicae* has been reported to promote aphid virulence upon overexpression in transgenic *Arabidopsis* lines by reducing the accumulation of 4‐methoxyindol‐3‐ylmethylglucosinolate, callose and hydrogen peroxide (H_2_O_2_) in response to aphid feeding (Elzinga *et al*., 2014). Transient overexpression of candidate effectors Me10 and Me23 of the potato aphid *Macrosiphum euphorbiae* enhances aphid performance, indicating their roles in the suppression of plant defence (Atamian *et al*., 2013). Me10 was recently reported to interact with tomato 14‐3‐3 isoform 7 (TFT7) to regulate plant susceptibility to aphids (Chaudhary *et al*., 2019). Transgenic barley plants expressing the candidate effector Rp1 of the bird‐cherry aphid *Rhopalosiphum padi* showed suppressed expression levels of jasmonic acid (JA), salicylic acid (SA) and ethylene (ET) defence‐related genes and significantly promoted aphid fecundity (Escudero‐Martinez *et al*., 2020).

The eliciting activity of aphid salivary proteins in plant defence responses was also detected. The infiltration of the watery saliva of *M. persicae* and the grain aphid *Sitobion avenae* induced plant defence responses and had adverse effects on aphid performance and feeding behaviour (De Vos and Jander, 2009; Zhang *et al*., 2017). Transient overexpression of Mp10 and Mp42 activated plant defence in *N. benthamiana* and reduced aphid fecundity (Rodriguez *et al*., 2014). In addition, Armet, a salivary protein of *A. pisum*, has been shown to induce an increase in SA accumulation by regulating the expression of *SAMT* and *SABP2*, which contribute to pathogen resistance in plants (Cui *et al*., 2019). The molecular chaperonin proteins derived from the aphid bacterial symbiont *Buchnera aphidicola*, GroEL of *A. pisum* and GroES of the grain aphid *Sitobion miscanthi* were found to trigger plant defence responses (Chaudhary *et al*., 2014; Li *et al*., 2022a).

RNA interference (RNAi) is widely used for gene function research in insects. It was developed as a promising approach for pathogen and pest control (Chung *et al*., 2021). Among various double‐stranded RNA (dsRNA) delivery methods, the spray application of dsRNA formulations may be one of the most suitable and promising dsRNA delivery methods for pathogen and pest control. A transdermal dsRNA delivery system with star polyamine nanocarriers was successfully established, improving the dsRNA penetration of the body wall of insects and the efficiency of gene silencing through spray‐based application (Li *et al*., 2019, 2022b). The involvement of *vestigial* and *Ultrabithorax* genes in the wing development of *M. persicae* and *CYP6CY3* in the host adaptation of cotton aphid *Aphis gossypii* was demonstrated via nanocarrier‐mediated RNAi through spray application (Wei *et al*., 2021; Zhang *et al*., 2022). This indicates that this method could be applied to identify the roles of aphid salivary proteins in modulating aphid‐plant interactions. Moreover, the greenhouse application of nanocarrier‐delivered RNA pesticides targeting *V‐type proton ATPase subunits d* (*ATP‐d*) and *g* (*ATP‐g*) of *M. persicae* had a high control efficacy against aphids; this further shows the great potential of nanocarrier‐mediated RNAi in aphid control (Ma *et al*., 2022).

Although the roles of salivary effectors are an important topic in the study of aphid virulence, few studies have been performed to precisely identify the composition salivary proteins of the cereal aphid *S. graminum* and investigate the roles of potential effectors in wheat‐aphid interactions. In the present study, we combined RNA‐Seq transcriptome sequencing analysis and proteomic analysis of watery saliva to identify the secreted proteins in aphid saliva. Using a *Pseudomonas fluorescens* effector‐to‐host analyser (EtHAn)‐mediated delivery system, we found that the putative salivary effector Sg2204 suppresses basal immunity in wheat and promotes aphid performance. *Sg2204*‐silenced aphids induce stronger defence responses in wheat, and silencing of *Sg2204* and its homologues by nanocarrier‐mediated RNAi has deterrent effects on aphid performance, suggesting its essential role in aphid feeding as an effector.

## Results

### Identification of salivary proteins and candidate effectors of *S. graminum*

In total, 62 276 916 bp raw reads were acquired from salivary glands of *S. graminum* (SAR accession number: SRR18308524). After removing adapters, ambiguous nucleotides and low‐quality sequences, 61 594 644 bp clean reads (9.24 G clean reads) remained. Subsequently, the transcriptome of *S*. *graminum* salivary glands was de novo assembled using the short reads assembly program Trinity; these reads were then clustered into 14 628 unigenes (Tables S1 and S2). These transcripts ranged from 301 to over 15 093 bp with an average size of 1631 bp (Figures S1–S4). All sequences of the unigenes in this study are provided in Data S1.

The raw MS/MS data of saliva were analysed using the MaxQuant software suite (version 1.5.2.8) and searched against the *S. graminum* salivary gland transcriptomic database. A total of 76 proteins were identified from the aphid watery saliva using LC–MS/MS (Table S3). Among them, 43 proteins were successfully annotated, with 33 proteins uncharacterized. Some digestive and detoxification enzymes were detected in aphid saliva, such as peroxidase and lipase. Several candidate effectors previously characterized in other aphid species were also identified in *S. graminum* saliva, including glucose dehydrogenase, C002 and Me10.

**Figure 1:**
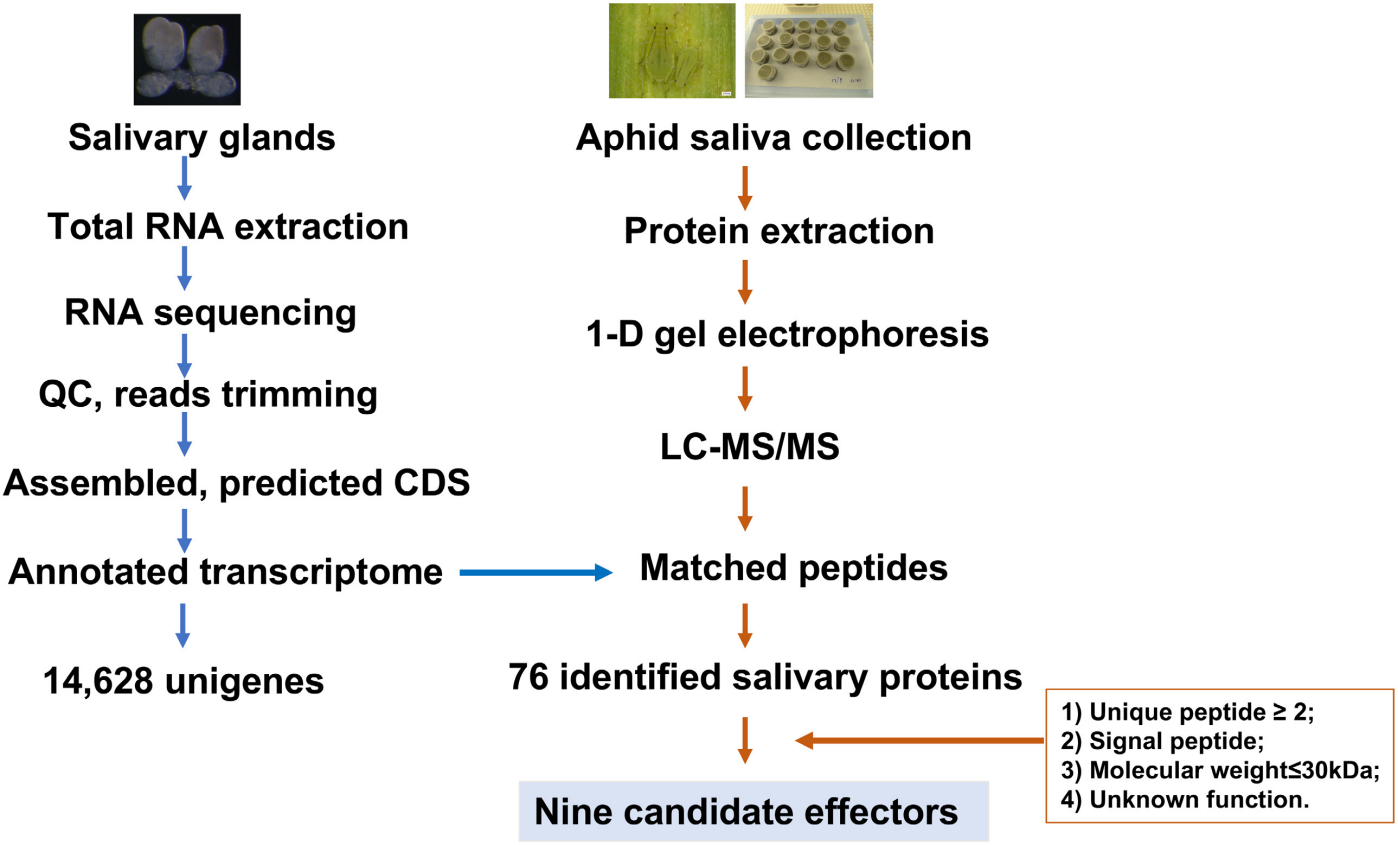
Schematic diagram of the bioinformatic analysis for the identification of salivary proteins and candidate effectors from the greenbug *Schizaphis graminum*.

A specific set of criteria was used to prioritize the proteins and generate a list of candidate effectors (Figure 1). The criteria (molecular weight < 30 kDa, predicted to be secreted, number of unique peptides more than 2, functionally unknown) were based on the characteristics of the previously identified fungi and aphid candidate effectors. This process led us to identify nine candidate effectors from *S. graminum* saliva, designated Sg1350, Sg1625, Sg1655, Sg1670, Sg1695, Sg1762, Sg1920, Sg1993 and Sg2204, and all these proteins were functionally unknown (Table 1).

**Table 1:** List of nine candidate effectors identified from *S. graminum* saliva

Protein name	Transcript ID	NCBI accession number	Unique peptides	Signal peptide (aa)	Molecular weight (kDa)	Annotation
Sg1350	Cluster‐5206.1350	XP_001950433.1	5	1–20	18.24	Uncharacterized protein LOC100168922
Sg1625	Cluster‐5206.1625	XP_001951405.1	5	1–22	22.68	Uncharacterized protein LOC100166545
Sg1655	Cluster‐5206.1655	XP_022165725.1	2	1–21	14.90	Uncharacterized protein LOC111030505
Sg1670	Cluster‐5206.1670	BAH70796.1	12	1–21	30.20	ACYPI000490
Sg1695	Cluster‐5206.1695	XP_022172257.1	7	1–20	23.87	Uncharacterized protein LOC111035063
Sg1762	Cluster‐5206.1762	XP_022160825.1	5	1–16	29.92	Uncharacterized protein LOC111026940
Sg1920	Cluster‐5206.1920	/	8	1–22	20.32	/
Sg1993	Cluster‐5206.1993	XP_015373648.1	2	1–25	27.16	Uncharacterized protein LOC107168676
Sg2204	Cluster‐5206.2204	XP_003246292.1	3	1–25	16.71	Uncharacterized protein LOC100570017

‘/’ indicates that no homologues of Sg1920 were identified.

### Identification and characterization of candidate effector Sg2204 in *S. graminum*

The expression of the nine candidate effectors in the salivary glands, heads, thorax, abdomen and midguts of apterous aphids was detected using RT‐qPCR. The results showed that all nine candidate effectors were specifically expressed in aphid salivary glands (Figure S5). For example, the relative expression of *Sg1350* and *Sg2204* in the salivary gland was 838.85 ± 92.76 and 1952.08 ± 0.74 times higher than that in the whole body, respectively.

**Figure 2:**
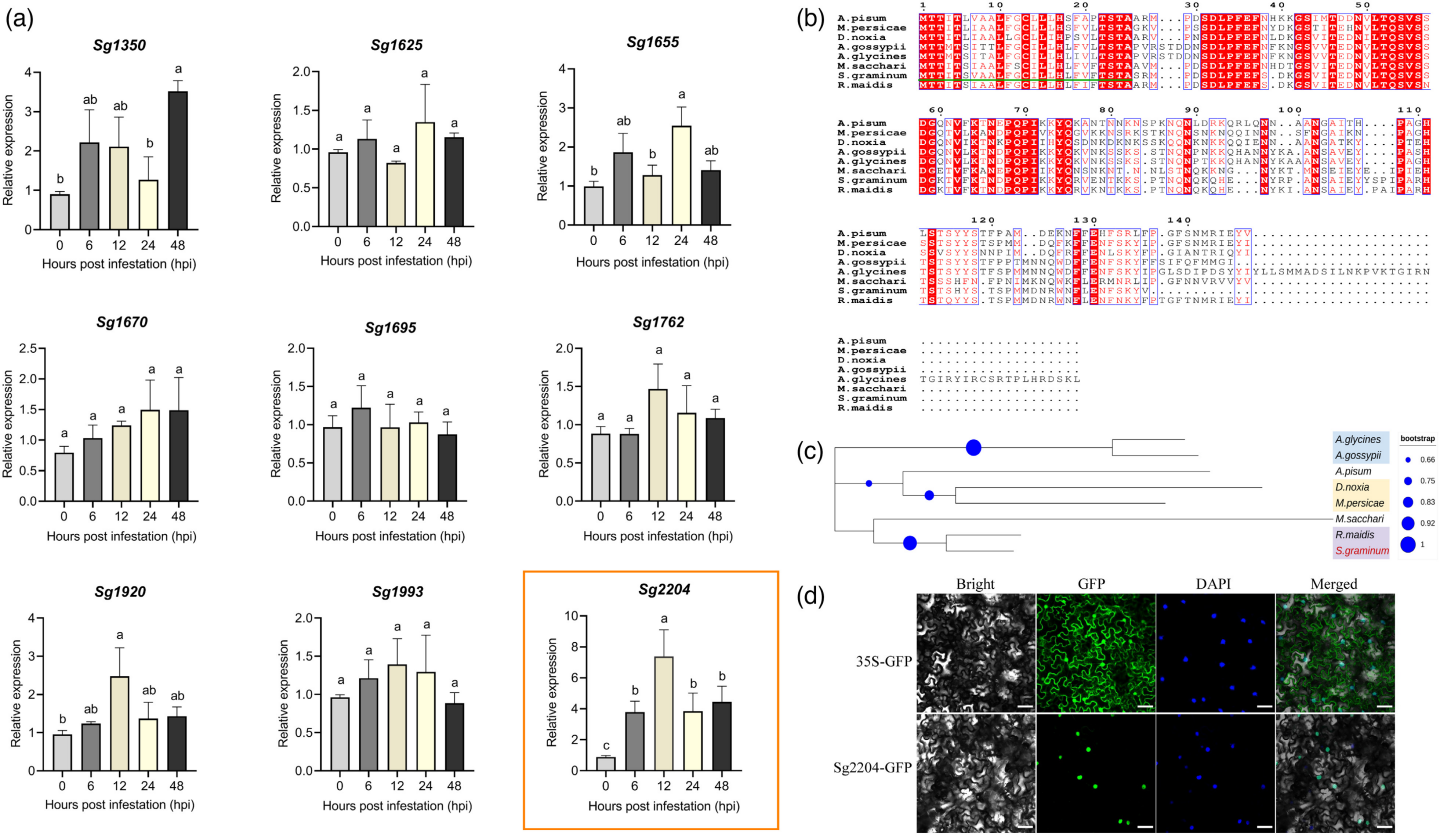
Characterization of Sg2204 in *Schizaphis graminum*. (a) RT‐qPCR results of the relative expression levels of nine candidate effectors of *S. graminum* after infestation on wheat plants at different time points. Data are presented as the mean ± SE (*n* = 3). Different lowercase letters above the bars indicate significant differences among groups (one‐way ANOVA followed by Duncan's multiple range tests, *P* < 0.05). (b) Multiple sequence alignment of the Sg2204 protein and orthologs from other aphid species. The deduced amino acid sequences from seven aphid species, including *Acyrthosiphon pisum* (NP_001156070.1), *Myzus persicae* (XP_022163343.1), *Diuraphis noxia* (XP_015374910.1), *Aphis gossypii* (XP_027851062.1), *Aphis glycines* (KAE9525059.1), *Melanaphis sacchari* (XP_025207447.1) and *Rhopalosiphum maidis* (XP_026819965.1). The sequence of *Sg2204* was submitted to NCBI GenBank under the accession number OM676656. Red shades indicate identical amino acids. Alignment was created using Clustal Omega. (c) Phylogenetic tree constructed by comparing the amino acid sequences of Sg2204 and the orthologs from other aphid species. A phylogenetic tree was constructed by the neighbour‐joining method using MEGA7.0. Bootstrap values calculated as a percentage for 1000 replications are shown at the nodes. The amino acid sequence with green underline indicates the signal peptide of the Sg2204 protein. (d) Subcellular localization of Sg2204. GFP (empty vector, pCAMBIA1300) and effector Sg2204 were transiently overexpressed in *N. benthamiana* leaves by *Agrobacterium* infiltration. The images were taken 3 days after agroinfiltration using confocal microscopy. Bar = 20 μm.

To further characterize the specific transcript profiles of these nine candidate effectors at different aphid feeding time points, the transcript levels of candidate effectors were analysed using RT‐qPCR. As shown in Figure 2a, the expression levels of *Sg1625*, *Sg1670*, *Sg1762*, *Sg1695* and *Sg1993* showed no significant changes during aphid infestation. The expression levels of *Sg1350*, *Sg1655* and *Sg1920* were slightly increased, with a 1.86–3.51‐fold change at some time points. However, the abundance of *Sg2204* was significantly highly induced during the infestation of wheat host plants and peaked at 12 hpi with an approximately 7.38‐fold increase. These results indicated that *Sg2204* was highly induced during aphid feeding and may contribute significantly to aphid virulence.

We obtained a full‐length cDNA for the *Sg2204* gene, which contains a 414‐bp open reading frame and encodes a polypeptide of 137 amino acid residues (Figure 2b). The first 25 amino acids constitute the signal peptide, with cleavage predicted between residues 25 and 26 (Figure 2b). BLAST analyses revealed that *Sg2204* has no strong matches to any protein of known function or to any predicted protein outside of the family Aphididae. The amino acid sequence of Sg2204 was aligned with the amino acid sequences of orthologs in seven other aphid species. The results showed that Sg2204 has the highest identity (90%) with the *R. madis* ortholog but only 65% and 55% sequence identity to *M. persicae* and *Diuraphis noxia* orthologs, respectively. Phylogenetic analysis showed that Sg2204 and the *R. madis* ortholog were closely related, clustering into an independent clade (Figure 2c).

To identify the subcellular localization of Sg2204, transient expression analyses of the Sg2204‐GFP fusion protein in *N. benthamiana* leaves were performed. The control groups expressing only GFP with the cauliflower mosaic virus 35S promoter exhibited fluorescence throughout the cytoplasm and nucleus (Figure 2d). But the fluorescence of the Sg2204‐GFP fusion protein was only observed in the nucleus of *N. benthamiana* cells, which suggested that Sg2204 encodes a nucleus‐targeting protein.

### Overexpression of Sg2204 in *Nicotiana benthamiana* suppresses cell death

**Figure 3:**
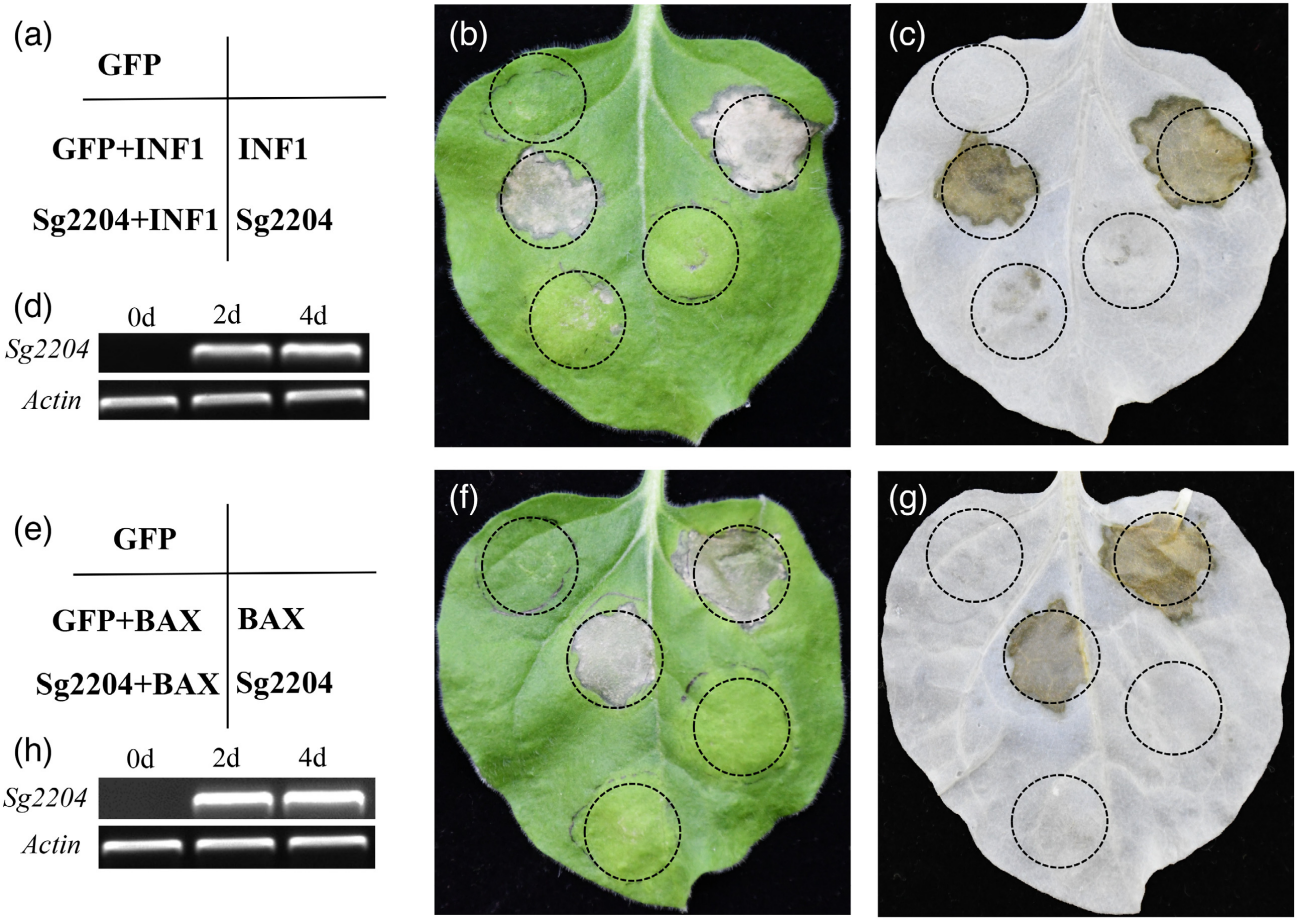
Transient overexpression of Sg2204 in *Nicotiana benthamiana* suppressed programmed cell death triggered by PAMP‐INF1 (a–d) and BAX (e–h). *N. benthamiana* leaves were infiltrated with recombinant strains of *Agrobacterium tumefaciens* cells carrying GFP (negative control) or Sg2204, and *A. tumefaciens* cells carrying INF1 (a, b) or BAX (e, f) were then infiltrated into the same region of leaves after 12 h, respectively. The leaves were decolorized with ethanol (c, g) and photographed 5 days after infiltration. Detection of the transcripts of *Sg2204* was performed using RT‐PCR in transient‐overexpressing *N. benthamiana* leaves inoculated with *A. tumefaciens* carrying Sg2204 at 2 and 4 days, and the reference gene *β‐actin* of *N. benthamiana* served as a control (d, h). All experiments were performed with three biological replications.

The potential roles of Sg2004 in the suppression of BAX and PAMP INF1‐induced programmed cell death (PCD), which resembles the defence‐related HR in plant cells, were examined. As shown in Figure 3a–g, transient overexpression of GFP and Sg2204 did not induce PCD, but overexpression of both BAX and INF1 in tobacco activated obvious PCD and overexpression of Sg2204 could significantly suppress BAX and INF1‐induced PCD in *N. benthamiana*. As a negative control, overexpression of GFP did not affect the induced PCD symptoms.

### Delivery of Sg2204 in wheat suppresses plant defence

**Figure 4:**
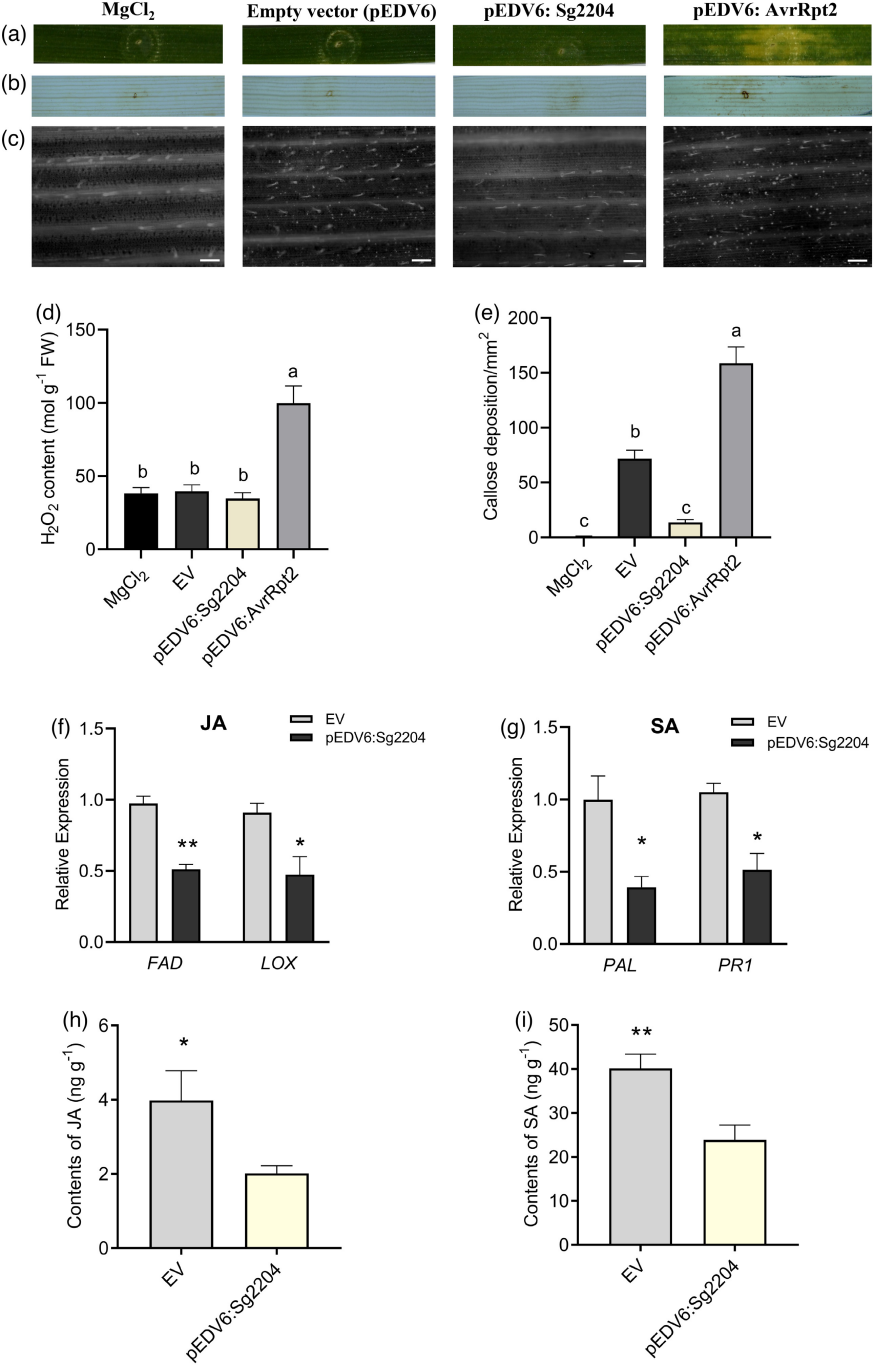
Delivery of Sg2204 into wheat suppressed plant defence. (a) Phenotypes of wheat leaves inoculated with MgCl_2_ buffer (mock), *Pseudomonas fluorescens* EtHAn carrying pEDV6 (empty vector, EV), pEDV6:*Sg2204* and pEDV6:*AvrRpt2* (positive control) at 72 h after infiltration. (b) DAB staining for H_2_O_2_ accumulation in wheat leaves infiltrated with MgCl_2_ buffer, *P. fluorescens* EtHAn carrying EV, pEDV6:*Sg2204* and pEDV6:*AvrRpt2* at 72 h after infiltration. (c) Callose deposition observed by fluorescence microscopy in wheat leaves infiltrated with MgCl_2_ buffer, *P. fluorescens* EtHAn carrying EV, pEDV6:*Sg2204* and pEDV6:*AvrRpt2* at 72 h after infiltration after aniline blue staining. Bars = 200 μm. (d) H_2_O_2_ content of wheat leaves inoculated with MgCl_2_ buffer, EV, pEDV6:*Sg2204* or pEDV6:*AvrRpt2*. (e) Average number of callose deposits/mm^2^ in wheat leaves treated with MgCl_2_ buffer, EV, pEDV6:*Sg2204* or pEDV6:*AvrRpt2*. Three biological replicates were performed for each treatment. Data are presented as the mean ± SE. Different lowercase letters above the bars indicate significant differences among groups (one‐way ANOVA followed by Duncan's multiple range tests, *P* < 0.05). (f) The expression levels of JA synthesis‐related genes *FAD* and *LOX* in wheat leaves after infiltration with *P. fluorescens* EtAnH carrying an empty vector (EV) or pEDV6:*Sg2204* at 72 h. (g) The expression levels of the SA synthesis‐related gene *PAL* and the SA signalling pathway marker gene *PR1* in wheat leaves after infiltration with an empty vector (EV) or pEDV6:*Sg2204* at 72 h. (h) Change in endogenous JA contents in wheat leaves treated with *P. fluorescens* EtAnH carrying an empty vector (EV) or pEDV6:*Sg2204*. (i) Change in endogenous SA contents in wheat leaves inoculated with *P. fluorescens* EtAnH carrying an empty vector (EV) or pEDV6:*Sg2204*. Three biological replicates were performed for each treatment. Data are presented as the mean ± SE. Asterisks above the bars indicate significant differences between controls and treatments (*P < 0.05; **P < 0.01; Student's *t* test).

To investigate the function of Sg2204 in inhibiting host plant immunity, *Sg2204* was cloned into the expression vector pEDV6 and then delivered into wheat leaves using the *P. fluorescens* EtAnH‐mediated delivery system. Previous studies reported that infiltration of EtHAn expressing AvrRpt2, a well‐characterized TTSS effector from *Pseudomonas syringae* was sufficient to induce in planta cell death and noticeable chlorotic phenotype in wheat leaves (Mindrinos *et al*., 1994; Mudgett and Staskawicz, 1999; Yin and Hulbert, 2011). As presented in Figure 4a,b, the wheat leaves infiltrated with EtAnH delivering the AvrRpt2 (positive control) showed obvious chlorosis symptoms and H_2_O_2_ accumulation after 2 days, suggesting that the T3SS system was successfully established in this study. The infiltration of EtAnH expressing Sg2204 did not induce any obvious chlorosis symptoms or H_2_O_2_ accumulation on the leaves. The H_2_O_2_ content in wheat leaves treated with AvrRpt2 was significantly increased compared with that in the control groups, but the H_2_O_2_ content in pEDV6 (empty vector)‐ and pEDV6:*Sg2204*‐inoculated wheat leaves was not significantly different from that in the control groups (Figure 4d).

As a nonpathogen, *P. fluorescens* has been shown to induce PTI‐related defence genes and callose deposition following infiltration into the leaves of several plant species (Nguyen *et al*., 2010). Aniline blue staining showed that many callose deposits were observed in wheat leaves challenged by *P. fluorescens* EtHAn carrying the pEDV6 empty vector and positive control pEDV6:*AvRpt2*, indicating the activation of PTI in wheat. However, significantly less callose deposition was detected in wheat leaves delivering Sg2204 (Figure 4c–e). Taken together, these results suggested that Sg2204 suppressed PTI‐related callose deposition in wheat plants.

Considering that the SA‐ and JA‐ pathways are major signalling pathways involved in most plant defences against insect herbivores, the key genes associated with SA and JA and the levels of plant defence hormones were studied upon delivery of Sg2204 in wheat for 2 and 4 days. Our results showed that the transcript levels of the SA biosynthesis enzyme *PAL* and SA pathway marker gene *PR1* were significantly down‐regulated after treatment with Sg2204 at 2 and 4 days, and the transcript levels of *FAD* and *LOX*, which are involved in the JA signalling pathway, were also significantly reduced at 4 days post‐infiltration of *P. fluorescens* EtAnH carrying Sg2204 (Figure 4f,g). Furthermore, LC–MS/MS results showed that delivery of Sg2204 in wheat significantly reduced the levels of JA and SA at 4 dpi compared with the control, suggesting that Sg2204 is involved in the suppression of both the SA and JA defence pathways in wheat plants (Figure 4h,i).

### Delivering Sg2204 into wheat promotes aphid feeding behaviour and enhances aphid performance

**Figure 5:**
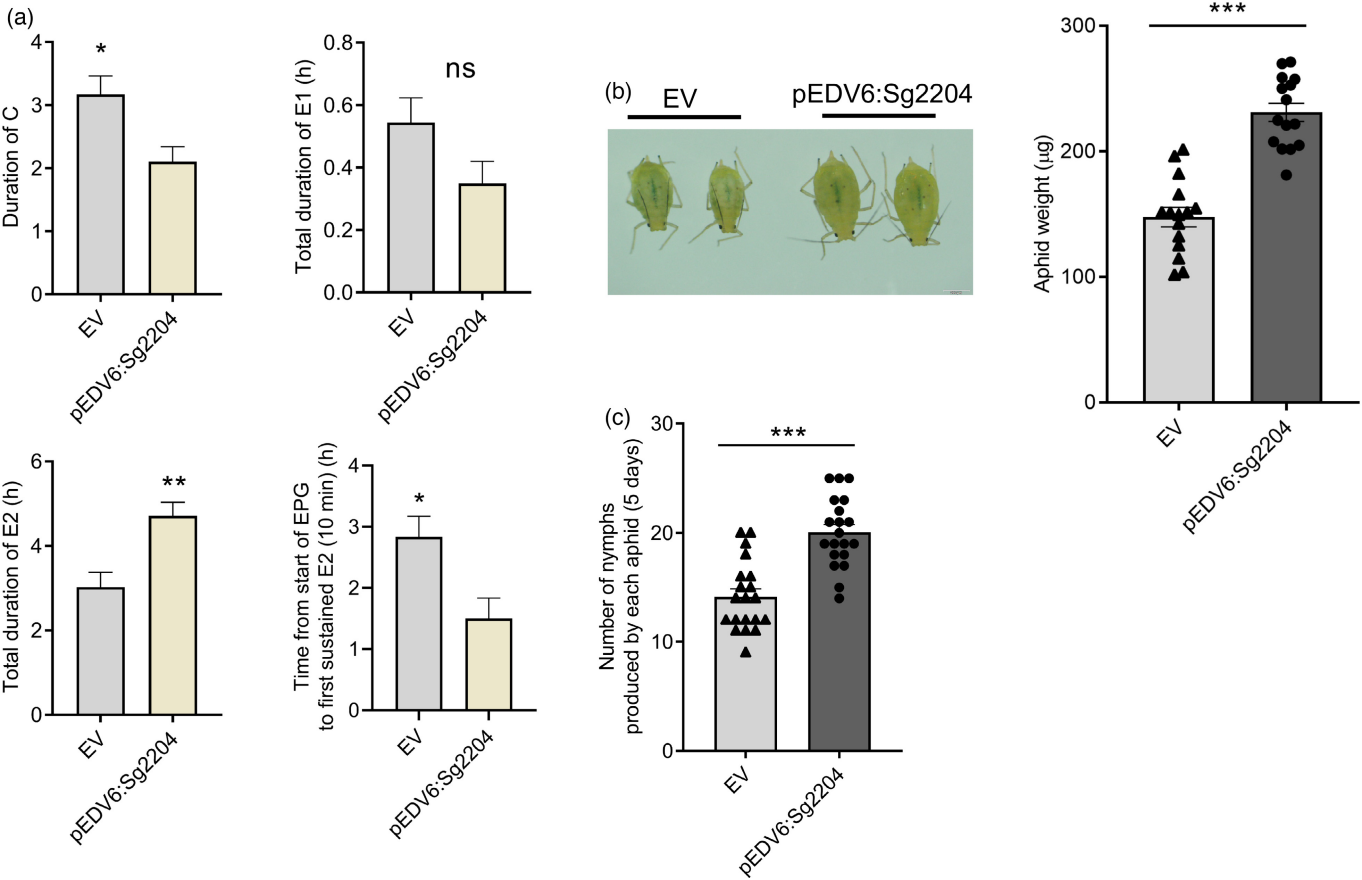
Overexpression of Sg2204 in wheat promoted *Schizaphis graminum* feeding and enhanced aphid body weight and fecundity. (a) Feeding behaviour parameters of *S. graminum*, including durations of stylet probing (C), salivation (E1), phloem ingestion (E2) and time from the start of EPG to the first sustained E2, when feeding on wheat leaves treated with empty vector (EV) and pEDV6:Sg2204. At least 10 biological replicates were performed for each group. (b) Body weight of *S. graminum* after feeding on wheat leaves inoculated with EV and pEDV6:*Sg2204* for 7 days (*n* = 15). (c) Number of nymphs produced by *S. graminum* after feeding on wheat leaves infiltrated with EV and pEDV6:*Sg2204* for 5 days (*n* = 20). Data are presented as the mean ± SE. Asterisks above the bars indicate significant differences between controls and treatments (*P < 0.05; **P < 0.01; ***P < 0.001; Student's *t* test).

As shown in Figure 5a, treating wheat with Sg2204 had a significant impact on the feeding behaviour of aphids. The duration of phloem ingestion (E2) was significantly higher in wheat treated Sg2204 compared with that infiltrated with the empty vector (control). In contrast, the durations of stylet probing activity (C) and nonprobing (Np) waveforms in Sg2204‐treated leaf areas were significantly higher than those in the control group. Additionally, aphids had a shorter latency to reach the first sustained E2 (>10 min) from the start of EPG recording when feeding on Sg2204‐infiltrated wheat leaves.

We found that the weight of aphids feeding on wheat leaves treated with Sg2204 was significantly greater than that on the control leaves (Figure 5b). The number of nymphs produced by aphid adults was also significantly higher on Sg2204‐treated leaves than on the control leaves (Figure 5c). These results suggested that Sg2204 enhanced aphid performance and host susceptibility by promoting aphid feeding.

### Sg2204 homologues from other aphid species have conserved functions in promoting wheat susceptibility to aphids

**Figure 6:**
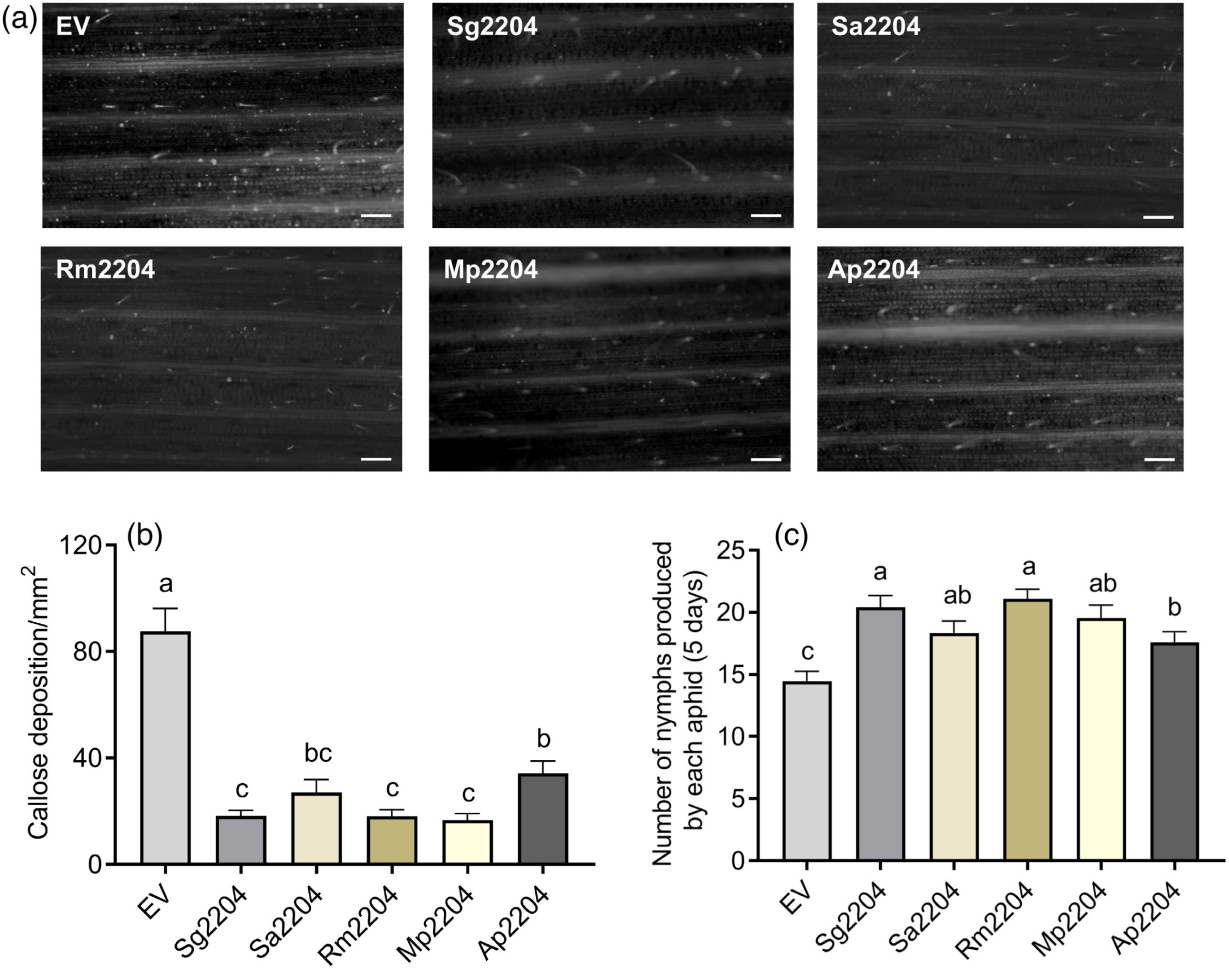
Sg2204 orthologs from *Sitobion avenae* (Sa2204) (NCBI accession no. OM831273), *Rhopalosiphum maidis* (Rm2204), *Myzus persicae* (Mp2204) and *Acyrthosiphon pisum* (Ap2204) have conserved functions in inhibiting wheat PTI defence and prompting aphid performance. (a) Callose deposition staining in wheat leaves after infiltration with *Pseudomonas fluorescens* EtHAn carrying an empty vector (EV), Sg2204, Sa2204, Rm2204, Mp2204 and Ap2204 at 2 days. Bars = 200 μm. (b) Average number of callose deposits/mm^2^ in wheat leaves inoculated with EV, Sg2204, Sa2204, Rm2204, Mp2204 and Ap2204 at 2 days (*n* = 3). (c) Number of nymphs produced by *Schizaphis graminum* after feeding on wheat leaves infiltrated with EV, Sg2204, Sa2204, Rm2204, Mp2204 and Ap2204 for 5 days (*n* = 15). Data are presented as the mean ± SE. Different lowercase letters above the bars indicate significant differences among groups (one‐way ANOVA followed by Duncan's multiple range tests, *P* < 0.05).

To investigate whether homologues of Sg2204 have conserved roles in suppressing PTI and promoting host susceptibility, Sg2204 and its homologues found in four other aphid species, *S. avenae* (Sa2204), *R. midis* (Rm2204), *M. persicae* (Mp2204) and *A. pisum* (Ap2204), were infiltrated into wheat using the *P. fluorescens* EtAnH‐mediated delivery system. As presented in Figure 6a,b, Sg2204 and all homologues inhibited callose deposition in treated wheat leaves compared with the control, and the number of callose deposits in wheat leaves infiltrated with the Sg2204 homologues was significantly less than that induced by nonpathogenic bacteria *P. fluorescens* carrying the empty vector. Furthermore, the results presented in Figure 6c show that the number of nymphs produced by *S. graminum* feeding on *Sg2204* homologue‐treated wheat leaves was significantly higher than those feeding on the control, indicating that *Sg2204* homologues have conserved functions in suppressing plant PTI and promoting aphid performance.

### Silencing of *Sg2204* by a nanocarrier‐mediated dsRNA delivery system reduces aphid performance and feeding

**Figure 7:**
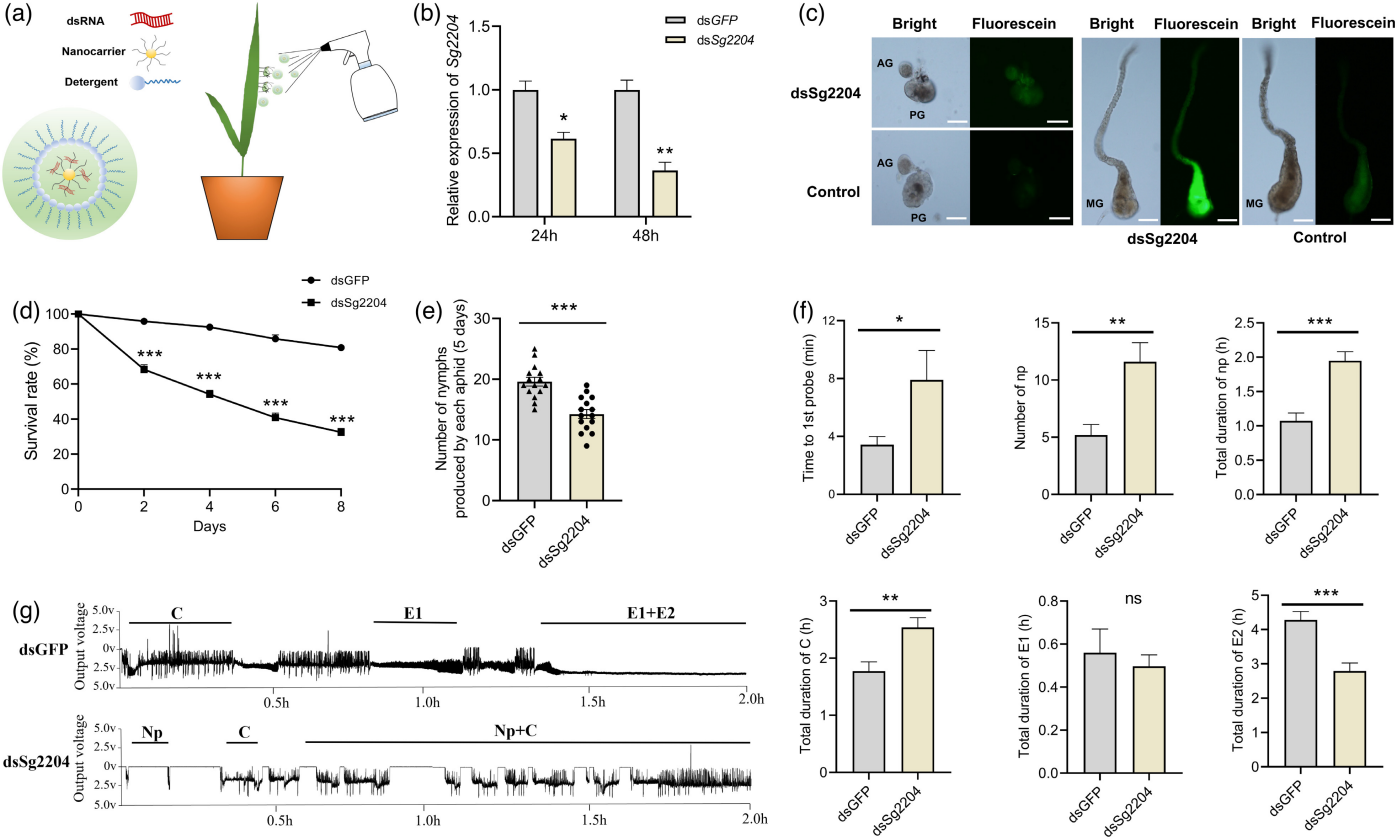
Silencing of *Sg2204* by nanocarrier‐mediated RNAi negatively affected aphid performance and feeding behaviour. (a) Schematic diagram of the spray method of application of the nanocarrier‐mediated transdermal dsRNA delivery system. (b) Relative expression levels of *Sg2204* at 24 and 48 h after dsSg2204/nanocarrier/detergent or dsGFP/nanocarrier/detergent treatment. Three biological replicates were conducted for each treatment (*n* = 3, Student's *t* test). (c) Examination of dsRNA uptake efficiencies in the salivary glands and guts of *S. graminum* using fluorescein‐labelled Sg2204‐dsRNA. Images were acquired at 12 h after spraying with fluorescein‐labelled Sg2204‐dsRNA/nanocarrier/detergent or H_2_O/nanocarrier/detergent (control) formulations using a fluorescence microscope. AG: accessory gland, HG: hindgut, MG: midgut and PG: principal gland. Scale bars = 100 μm. (d) Survival rate of dsGFP‐ or dsSg2204‐treated *S. graminum* after feeding on wheat plants at various time points (*n* = 10, Student's *t* test). (e) Number of nymphs produced by dsGFP‐ or dsSg2204‐treated *S. graminum* after feeding on wheat plants for 5 days (*n* = 15, Student's *t* test). (f) Effects of *Sg2204* RNAi knockdown on the feeding behaviours of *S. graminum* based on EPG recordings (*n* ≥ 10, Mann–Whitney *U* test). (g) Representative EPG waveforms of *Sg2204*‐silenced aphids (dsSg2204) and control aphids (dsGFP) on wheat plants. Np, nonprobing; C, stylet probing; E1, salivation and E2, phloem ingestion. The data shown are the mean ± SE. Asterisks above the bars indicate significant differences between controls and treatments (ns, not significant; *P < 0.05; **P < 0.01; ***P < 0.001).

A schematic diagram of the transdermal dsRNA delivery system with nanocarriers is shown in Figure 7a, and the dsRNA formulation was sprayed onto aphids and wheat plants. After 12 h of treatment, obvious labelled‐dsRNA fluorescence was detected in the salivary glands and guts of aphids (Figure 7c). The RNAi efficiency of nanocarrier‐mediated RNAi on the *Sg2204* gene was detected using RT‐qPCR. As shown in Figure 7b, the expression levels of *Sg2204* in *S. graminum* were reduced significantly after treatment with 200 ng/μL dsSg2204 for 24 h. After 48 h of treatment, the transcript levels of *Sg2204* were further decreased to 0.36 ± 0.065‐fold, which were significantly lower than those in the control.

The effects of silencing *Sg2204* on aphid survival, fecundity and feeding behaviour were also investigated. The results in Figure 7d,e show that the survival rate of *S. graminum* treated with dsSg2204 was reduced to 54% after feeding on wheat plants for 4 days, which was significantly lower than that of the control group (dsGFP); this rate further decreased to 32% at 8 days. In addition, the number of nymphs produced by *Sg2204*‐silenced aphids was also significantly less than that produced by aphids in the control groups.

Aphid feeding behaviour was negatively affected after the expression of *Sg2204* was inhibited. As shown in Figure 7f,g, the time to the first probe activity of aphids treated with dsSg2204 was significantly longer than that spent of the control. The number of nonprobing waveforms of *Sg2204*‐silenced aphids on wheat plants was greater than that on the control. In addition, the durations of the nonprobing and C phases of aphids treated with dsSg2204 were longer, but the duration of phloem ingestion (E2) was shorter, than those of aphids treated with dsGFP.

### *Sg2204*‐silenced aphids activate stronger wheat defence responses

**Figure 8:**
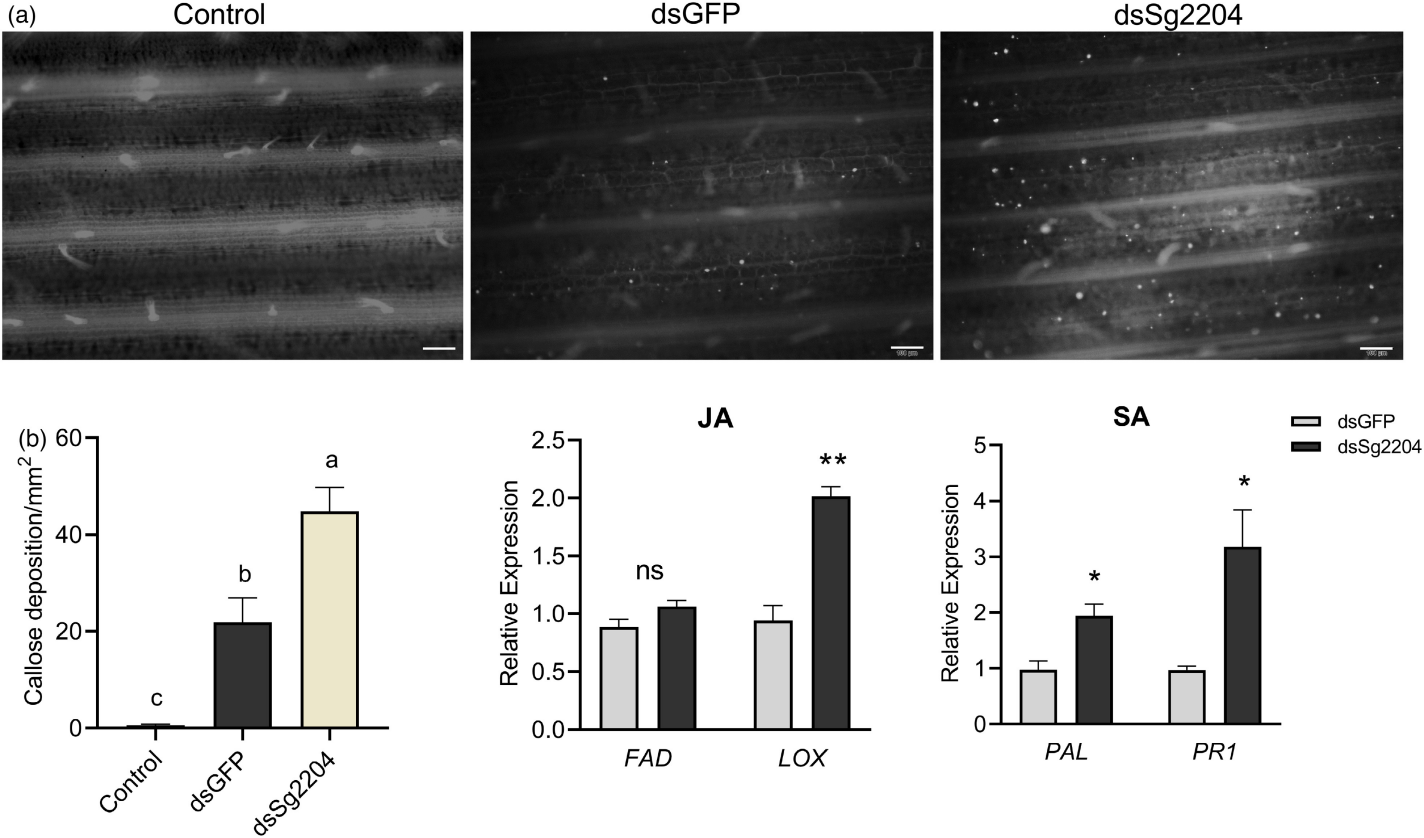
Infestation with *Sg2204*‐silenced *Schizaphis graminum* induced a stronger wheat defence response. (a) Callose deposition staining in wheat leaves after infestation with no aphids (control), dsGFP‐treated aphids or dsSg2204‐treated aphids. Bars = 200 μm. (b) Average number of callose deposits/mm^2^ in wheat leaves infested with no aphids (control), dsGFP‐treated aphids or dsSg2204‐treated aphids. (c) Relative expression levels of the JA‐related genes *FAD* and *LOX* and the SA marker genes *PAL* and *PR1* in wheat leaves infested with Sg2204‐silenced aphids (dsSg2204) or control aphids (dsGFP). The data shown are the mean ± SE. Three biological replicates were performed for each group. Asterisks above the bars indicate significant differences between controls and treatments (ns, not significant; *P < 0.05; **P < 0.01; ***P < 0.001; Student's *t* test).

As shown in Figure 8a,b, we found that more callose was deposited in wheat leaves induced by *Sg2204*‐silenced aphid feeding compared with the control (without aphid infestation) and treatment groups induced by dsGFP‐fed aphids. The changes in the expression levels of the JA biosynthesis pathway‐associated genes *FAD* and *LOX* and the SA biosynthesis/signalling pathway‐related genes *PAL* and *PR1* were also examined in wheat leaves infested with *Sg2204*‐silenced *S. graminum* using RT‐qPCR. As shown in Figure 8c, the expression levels of *LOX*, *PAL* and *PR1* in wheat leaves infested with *Sg2204*‐silenced aphids were significantly higher than those in wheat leaves infested with aphids fed dsGFP.

### Silencing of *Sg2204* homologues from other aphid species through a nanocarrier dsRNA delivery system reduces aphid survival and fecundity

**Figure 9:**
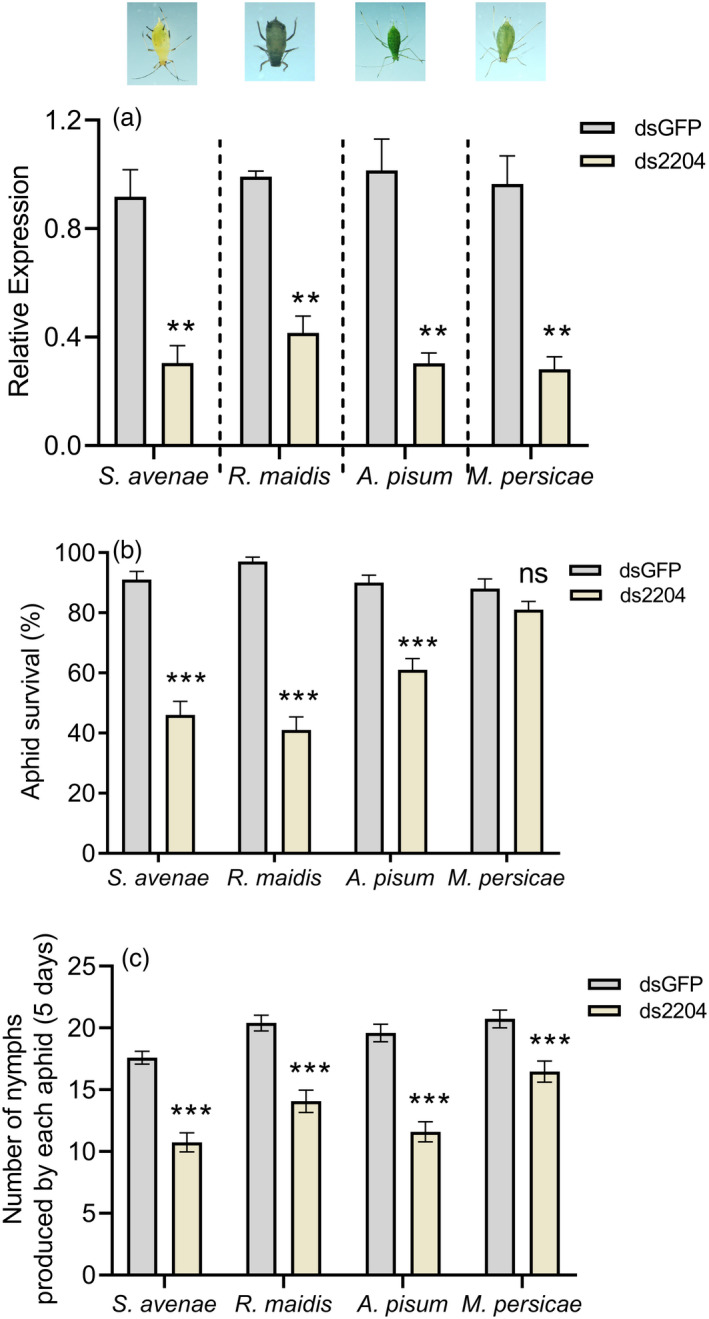
Silencing of *Sg2204* homologues from *Sitobion avenae*, *Rhopalosiphum maidis*, *Acyrthosiphon pisum* and *Myzus persicae* via a transdermal dsRNA delivery system with nanocarriers decreased aphid survival and fecundity. (a) Relative expression levels of *Sg2204* homologues after treatment with ds2204/nanocarriers/detergent or dsGFP/nanocarriers/detergent at 48 h (*n* = 3, Student's *t* test). (b) Survival rate of ds2204‐ or dsGFP‐treated *S. avenae*, *R. maidis*, *A. pisum* and *M. persicae* at 6 days after feeding on wheat, maize, broad bean and tobacco plants, respectively (*n* = 10, Student's *t* test). (c) Number of nymphs produced by ds2204‐ or dsGFP‐treated *S. avenae*, *R. maidis*, *A. pisum* and *M. persicae* when feeding on wheat, maize, soybean and tobacco plants for 5 days (*n* = 15, Student's *t* test). Data are presented as the mean ± SE. Asterisks above the bars indicate significant differences between controls and treatments (ns, not significant; *P < 0.05; **P < 0.01; ***P < 0.001; Student's *t* test).

Using the spray application method for nanocarrier‐based transdermal dsRNA delivery system, the expression levels of *Sg2204* homologues from *S. avenae*, *R. maidis*, *A. pisum* and *M. persicae* were all significantly down‐regulated, which decreased to 28%–41% of that of control groups (*P* < 0.01) (Figure 9a). Additionally, RNAi‐mediated silencing of *Sg2204* homologues in aphids significantly reduced the survival and fecundity of *S. avenae*, *R. maidis* and *A. pisum* (*P* < 0.001), and the percentages of aphid survival after gene silencing varied among different species. For example, the survival rates of *A. pisum* were reduced to 61% after gene silencing, but the survival rate of *R. maidis* was only 41%. However, gene silencing had no significant impacts on the survival of *M. persicae* (*P* = 0.052*)*, but it significantly reduced the fecundity of four aphid species (*P* < 0.001) (Figure 9b,c).

## Discussion

Due to the poor efficiency of *Agrobacterium*‐mediated transformation in cereal plants, few studies have been reported on the roles of salivary proteins from cereal aphids in modulating plant defence. The greenbug *S. graminum* is a major phytotoxic aphid pest of wheat, sorghum and barley (Blackman and Eastop, 2000); therefore, it is crucial to identify and functionally characterize salivary effectors from *S. graminum* to uncover the mechanisms employed by aphids when infesting cereals.

In our study, a total of 76 *S. graminum* watery salivary proteins were identified using integrated transcriptomic and proteomic analysis. Consistent with a previous study (Nicholson and Puterka, 2014), the presence of several proteins, such as glucose dehydrogenase, peroxidase, glutathione peroxidase and carbonic anhydrase, was also detected in *S. graminum* saliva in our study. We also identified several salivary proteins of *S. graminum* that had sequence similarity to several previously identified putative aphid effectors. For example, two salivary proteins with similarity to effector C002 from *M. persicae* and Me10 from *M. euphorbiae* were detected in *S. graminum* saliva. These proteins have previously been shown to suppress plant defence and promote aphid virulence (Atamian *et al*., 2013; Bos *et al*., 2010). In addition, we also identified cathepsin B protein in *S. graminum* saliva, which has been demonstrated to be involved in eliciting plant defence as an elicitor of *M. persicae* and to affect host adaptability (Guo *et al*., 2020). Whether these candidate effectors of *S. graminum* display conserved functions in modulating plant defence remains to be explored.

Delivering candidate effectors into cereal plants, such as wheat or barley, using the *P. fluorescens* EtHAn‐mediated delivery system has been successfully applied for the functional characterization of effectors from the wheat stripe rust fungus *P. striiformis* f. sp. *tritici* (Bai *et al*., 2021; Cheng *et al*., 2017; Upadhyaya *et al*., 2014; Xu *et al*., 2019a). In our study, delivery of Sg2204 into wheat using the *P. fluorescens* EtHAn system enhanced aphid fecundity and body weight and promoted aphid phloem feeding. Some researchers have proven that the salivary effectors of aphids and other hemipteran insects enhance host susceptibility by modulating plant defence pathways. For example, salivary effector Bt56 of *Bemisia tabaci* interacting with NTH202 promotes the susceptibility of tobacco to whitefly by affecting SA‐mediated plant defences (Xu *et al*., 2019b). Salivary proteins, such as BtFer1 from *B. tabaci*, glutathione peroxidase from the mirid bug *Apolygus lucorum* and vitellogenin from the small brown planthopper *Laodelphax striatellus*, attenuate H_2_O_2_‐mediated plant defence and improve insect feeding performance as effectors (Dong *et al*., 2020; Ji *et al*., 2021; Su *et al*., 2019).

A previous study demonstrated that callose deposition induced by aphids is involved in the sealing of sieve pores as a phloem defence mechanism that prevents the flow of nutrients to piercing‐sucking insects (Will and van Bel, 2006). In addition, the plant defence hormones JA and SA are important signalling molecules conferring plant resistance against aphids (Li *et al*., 2006; Yates and Michel, 2018; Züst and Agrawal, 2016). The expression levels of genes involved in both the JA‐ and SA‐dependent defence pathways of wheat, such as *LOX*, *FAD*, *PAL* and *PR1*, are significantly up‐regulated in response to *S. graminum* infestation (Reddy *et al*., 2013; Zhang *et al*., 2020; Zhu‐Salzman *et al*., 2004). Exogenous application of SA and JA on *Arabidopsis*, wheat, tomato and *Brassica* plants had negative effects on aphid population growth (Ali *et al*., 2021; Boughton *et al*., 2006; Bruce *et al*., 2003; Feng *et al*., 2021). Our results showed that delivery of Sg2204 into wheat suppressed callose deposition and JA‐ and SA‐dependent defence responses induced by nonpathogenic *P. fluorescens*. Therefore, the effects of Sg2204 on enhanced plant susceptibility to *S. graminum* are proposed to be associated with the suppression of both the JA‐ and SA‐dependent plant defence signalling pathways in wheat. Although an antagonistic relationship between SA and JA signalling pathways is evident (Thaler *et al*., 2012), some components of plants can activate or suppress the SA and JA signalling pathways simultaneously. For example, the expression of a mannose‐specific JRL‐like gene (TaJRLL1) of wheat in *Arabidopsis thaliana* increased the levels of both JA and SA and enhanced its resistance against pathogens (Xiang *et al*., 2011). WRKY70, a transcriptional factor, inhibited plant immunity by suppressing the expression of SA and JA signalling pathway genes, such as *NPR1*, *VSP1* and *VSP2* (Chakraborty *et al*., 2020; Despres *et al*., 1995; Li *et al*., 2004). It is speculated that Sg2204 interacts with the components of wheat plants that are involved in modulating both JA and SA defensive pathways. A previous study also showed that transgenic barley lines expressing the *R. padi* effector Rp1 reduced expression levels of JA‐, SA‐ and ET‐responsive genes and had enhanced plant susceptibility to *R. padi* (Escudero‐Martinez *et al*., 2020).

We also found that Sg2204 orthologs from five other aphid species inhibited wheat defence and promoted aphid fecundity, suggesting the functional conservation of the effector in Aphidinae. A previous study showed that *M. persicae* effector Mp10 orthologs from diverse plant‐feeding hemipteran insects have evolved conserved functions for suppressing ROS and Ca^2+^‐burst in *N. benthamiana* leaf discs upon elicitation with flg22 (Drurey *et al*., 2019). In addition, the *M. euphorbiae* effector Me10 and its homologue in *A. gossypii*, Ag10k, have been proven to interact with TFT7 in planta and interfere with the function of the TFT7 immune complex to promote aphid virulence (Chaudhary *et al*., 2019). However, the aphid protein effector C002 was previously reported to promote aphid colonization in a plant species‐specific manner, likely due to the presence or lack of NDNQGEE repeat motifs (Pitino and Hogenhout, 2013). Whether the Sg2204 orthologs from the other aphid species, which were not detected in this study, are involved in the suppression of host plant defence remains to be investigated.

Delivery of dsRNA into cells to silence the genes has been successfully applied to analyse the function of candidate effectors of hemipterans (Pitino *et al*., 2011; Xu *et al*., 2019b). Nanocarrier‐mediated RNAi is a practical delivery system for silencing the target genes of insects such as the soybean aphid *Aphis glycines* and *M. persicae* and is regarded as a promising strategy for gene function characterization and pest control (Li *et al*., 2022b; Niu *et al*., 2021; Yan *et al*., 2020). In this study, to further examine the roles of Sg2204 in suppressing plant defence, the *Sg2204* gene was silenced through spray application of a nanocarrier‐mediated transdermal dsRNA delivery system. Our results showed that the expression levels of *Sg2204* were significantly inhibited after the application of dsSg2204/nanocarriers/detergent, and its expression levels were reduced by more than 60% compared with those of the control groups. Previous study showed that the transcript levels of *ATP‐d* and *ATP‐g* in *M. persicae* decreased to 60%–70% after spray application of nanocarrier/dsRNA formulations (Ma *et al*., 2022), suggesting that the efficiency of nanocarrier‐mediated RNAi through spray method is stable in different aphid species. As expected, the expression levels of JA‐ and SA‐responsive genes were increased, and more callose deposits were observed in wheat after *Sg2204*‐silenced aphid infestation, which further suggested the involvement of Sg2204 in the suppression of plant basal defence. It was also found that the duration of nonprobing phase and stylet‐probing activity of aphids was significantly prolonged after silencing *Sg2204*. We cannot exclude the possibility that the mechanical damage caused by the frequent puncturing of the stylet lead to a stronger defence response of wheat. Interestingly, the silencing of *Sg2204* homologues from four aphid species had deterrent effects on aphid survival and fecundity, indicating that this gene has conserved functions in aphid adaptability to host plants. Aphid salivary proteins which are essential for the infestation of plants appear to be a promising target for RNAi‐mediated pest control. For example, the silencing of salivary protein C002 in *M. persicae* and structural sheath protein in *S. avenae* via host‐induced gene silencing (HIGS) led to a significant decline in the reproduction and survival rates of aphids (Abdellatef *et al*., 2015; Pitino *et al*., 2011). Thus, Sg2204 and its homologues could be used as ideal RNAi target genes for aphid control through HIGS and spray‐induced gene silencing (Ma *et al*., 2022; Qiao *et al*., 2021; Sun *et al*., 2019).

Additionally, subcellular localization showed that Sg2204 protein localized to the nucleus in *N. benthamiana*, suggesting that this effector may interact with nuclear proteins of wheat to inhibit plant immunity. Some researchers have demonstrated that effectors of Fusarium head blight *Fusarium graminearum* and *L. striatellus* could interact with proteins localized in the plant cell nucleus, such as the transcription factor WRKY71 and protein kinase TaSnRK1α28, to suppress plant defence (Ji *et al*., 2021; Jiang *et al*., 2020). Therefore, it is worth identifying the proteins that interact with Sg2204 in wheat plants using a yeast two‐hybrid system in future studies.

Overall, our findings provide the first evidence that *S. graminum* employs the effector protein Sg2204 to enhance aphid performance on wheat by suppressing JA‐ and SA‐related signalling pathways to manipulate plant defence responses. Our findings also demonstrate that silencing of *Sg2204* and its homologues by nanocarrier‐mediated RNAi was lethal to aphids. These results provide new insights into the molecular mechanisms underlying cereal aphid‐plant interactions and RNAi‐based strategies for aphid control.

## Experimental procedures

### Plants and insects

Clones of *S. graminum*, *S. avenae*, *A. pisum* and the corn aphid *Rhopalosiphum maidis* were initially established from a single aphid collected from a field in Langfang city, Hebei Province, northern China, and clones of *M. persicae* were established from a single aphid collected from a tobacco field in Kunming city, Yunan Province, southwestern China. All aphid clones were maintained under laboratory conditions (L : D = 16 h : 8 h; 20 ± 1 °C) on corresponding host plants, including wheat, broad bean, maize and tobacco plants for more than 4 years.

### RNA isolation and library construction

Approximately 500 paired salivary glands of alate and apterous adult aphids were dissected in cold phosphate‐buffered saline (pH 7.2; HyClone, Thermo Scientific, MA). Total RNA was isolated using TRIzol Reagent (Invitrogen, Carlsbad, CA) according to the manufacturer's protocol. RNA concentration and integrity were measured using a Qubit 2.0 Fluorometer (Life Technologies, Carlsbad, CA) and a Bioanalyzer 2100 system (Agilent Technologies, Santa Clara, CA), respectively. Total RNA samples (3 μg) with standard quality were used to generate the sequencing libraries with the NEBNext® UltraTM RNA Library Prep Kit for Illumina® (New England Biolabs, Beverly, MA) following the manufacturers' instructions. The library preparations were sequenced using an Illumina HiSeq 2500/MiSeq platform and paired‐end reads (the sequencing strategy was PE150). After removing adaptor sequences, ambiguous ‘N’ nucleotides and low‐quality sequences, the clean reads were assembled using Trinity r20140413p1 min_kmer_cov:2 and the other default parameters as described for de novo transcriptome assembly without a reference genome to generate transcripts and unigenes. The unigenes were searched against the nonredundant protein database (Nr), NCBI nucleotide database (Nt) and Swiss‐Prot protein database with an *E* value < 10^−5^ and against euKaryotic Ortholog Groups of proteins (KOG) with an *E* value < 10^−3^. The unigenes were annotated by Blast2GO in the Gene Ontology (GO) database with an *E*‐value threshold of 1.0^−6^ and annotated by the KEGG Automatic Annotation Server (KAAS) based on the Kyoto Encyclopedia of Genes and Genomes (KEGG) with an *E* value threshold of 1.0^−10^.

### Identification of aphid salivary proteins using liquid chromatography−mass spectrometry/mass spectrometry (LC–MS/MS)

*Schizaphis graminum* watery saliva was collected as described previously (Zhang *et al*., 2017). Proteins were electrophoresed on a 12.5% acrylamide separating gel with a 4% stacking gel for 1.5 h at 120 V. The gel was stained using the PlusOne Silver Staining Kit (GE Healthcare, Little Chalfont, Buckinghamshire, UK) following the manufacturer's instructions. Protein bands were excised from the prepared 1D gels and were then destained, reduced, alkylated and trypsin‐digested as described by Harmel *et al*. (2008). The shotgun analysis was conducted on an Easy nLC 1000 (Thermo Fisher Scientific, Waltham, MA) coupled with a Q Exactive mass spectrometer (Thermo Fisher Scientific, San Jose, CA). The raw MS data were analysed using the MaxQuant software suite (version 1.5.2.8) and searched against the *S. graminum* salivary gland transcriptomic database. For the initial search, the precursor ion mass tolerance was 15 ppm. The search followed the enzymatic cleavage rule for trypsin; a maximum of two missed cleavage sites and 20 mmu mass tolerance for the fragment ions were allowed. For database searching, cysteine carbamidomethylation was considered a static modification, while N‐terminal acetylation and methionine oxidation were considered dynamic modifications. The cutoff for the global false discovery rate (FDR) in the peptide‐spectrum match (PSM) and protein identification was <0.01.

### Subcellular localization

The coding sequence of *Sg2204* (without a signal peptide) was constructed in pCAMBIA1300 with TagGFP fused at the C‐terminus. All constructs, including the empty vector pCAMBIA1300, were transformed into *Agrobacterium tumefaciens* GV3101 and cultured overnight in LB medium (50 μg/mL kanamycin, 25 μg/mL rifampicin) at 28 °C. The cells were harvested and resuspended in infiltration medium (10 mM MgCl_2_, 10 mM 2‐(N‐morpholino) ethanesulphonic acid, 200 μM acetosyringone, pH 5.6) to an OD600 of 0.5. *Agrobacterium* carrying the effector‐GFP constructs were then infiltrated into the leaves of 6‐week‐old *N. benthamiana* plants using a 1‐mL syringe without a syringe needle. The infiltrated leaves were collected at 3 days after infiltration and observed under a Zeiss LSM 880 laser confocal microscope (Zeiss, Jena, Germany). Cell nuclei were stained with 4,6‐diamidino‐2‐phenylindole (DAPI). GFP was excited at 488 nm, and DAPI signals were analysed at 405 nm.

### *Agrobacterium tumefaciens* infiltration assays for the suppression of BAX/INF1‐induced programmed cell death

The coding sequence of *Sg2204* excluding signal peptide was cloned into pGR107, and *A. tumefaciens* GV3101 carrying pGR107‐Sg2204 constructs was infiltrated into *N. benthamiana* leaves as described above. *A. tumefaciens* cells carrying BAX/INF1 were infiltrated into the same site 24 h later. The photos of leaf symptoms were taken 3 days after inoculation with BAX/INF1. The leaves were decolorized using ethanol. Three biological replicates were performed for each treatment. *A. tumefaciens* cells carrying GFP were used as negative controls.

### Bacterial T3SS‐mediated overexpression in wheat plants

The coding sequence of *Sg2204* and its orthologs with the predicted signal peptide excluded and the final stop codon included were amplified using Phusion High‐Fidelity DNA Polymerase (Thermo Scientific, Waltham, MA) according to the manufacturer's instructions. The PCR products were inserted into the entry vector pDONER221 by BP cloning and then introduced into the Gateway destination vector pEDV6 by an LR clonase reaction according to the manufacturer's instructions. The inserted PCR products were confirmed by sequencing. The primers used in this study are listed in Table S4.

The pEDV6:*Sg2204* construct was transformed into the *P. fluorescens* strain EtHAn by electroporation. For the infiltration of the leaves, recombinant strains of EtHAn were grown in KB liquid medium (25 μg/mL rifampicin, 30 μg/mL gentamicin) for 24 h and resuspended in 10 mM MgCl_2_ solution. EtHAn suspensions (OD_600_ = 1.5) were infiltrated into the second leaves of wheat seedlings at the two‐leaf stage using a syringe without a needle. The infiltrated plants were grown and maintained in a climate chamber at 20 ± 1 °C for 2 days.

### Detection of callose deposition and H_2_O_2_ accumulation in wheat leaves

Callose deposition in wheat leaves was visualized according to previously reported protocols (Hood and Shew, 1996). Callose deposition was observed and photographed with an Echo Revolve Hybrid Microscope (Echo Laboratories, San Diego, CA) using a DAPI filter. Thirty sites (1 mm^2^/site) were selected from infiltrated areas of each treated leaf, and the number of callose deposits was counted from each site. Three independent biological replicates were conducted. H_2_O_2_ accumulation was examined in wheat leaves by 3′‐diaminobenzidine (DAB) staining according to the histochemical methods described by Zhao *et al*. (2018). The stained leaves were imaged using an Olympus SZX‐16 (Olympus Corporation, Tokoyo, Japan). The endogenous H_2_O_2_ content in the wheat leaves was determined using the protocols reported by Ferguson *et al*. (1983).

### RT‐qPCR analysis

The expression levels of candidate effector genes in aphid tissues and feeding periods on wheat plants were examined using RT‐qPCR analysis. RT‐qPCR was performed on an ABI 7500 Real‐Time PCR System (Applied Biosystems, Foster City, CA) as described previously (Zhang *et al*., 2017). Changes in the expression levels of genes involved in the JA and SA defence signalling pathways, including *FAD*, *LOX*, *PAL* and *PR1*, in wheat after infiltration with Sg2204 or infestation with Sg2204‐silenced aphids were detected. All primers for RT‐qPCR are presented in Table S4. All reactions were performed with three biological replicates, and the differential expression was calculated using the $2^{-\Delta \Delta C_{T}}$ method (Livak and Schmittgen, 2002).

### Measurement of phytohormones in wheat leaves

The phytohormones were analysed as described previously with some modifications (Xiang *et al*., 2011). In brief, 0.5 g of wheat leaves infiltrated with *P. fluorescens* EtHAn carrying Sg2204 or the empty vector at 2 days were harvested and homogenized in liquid nitrogen. Phytohormone extraction was performed by adding 10 mL of ethyl acetate, followed by ultrasound extraction for 20 min and centrifugation at 16000 g for 20 min at 4 °C. The supernatants were evaporated under a stream of nitrogen at 40 °C. The final extracts were redissolved in 1 mL of 70% methanol and centrifuged at 16000 g for 10 min. The supernatants were then collected and used as samples for further analysis with a liquid chromatography–tandem mass spectrometry system (LC–MS/MS). Three biological replicates were performed.

### Aphid performance on wheat infiltrated with *P. fluorescens* EtHAn carrying Sg2204 and its homologues

After 2 days of *P. fluorescens* EtHAn infiltration, 10 apterous adult aphids were transferred to the infiltration sites of wheat leaves and restricted to a clip cage (2.5 × 2.5 × 2.5 cm). The number of nymphs produced by the aphids on each plant was recorded daily for 5 days, and 15 replicates were conducted in each group. Ten newborn nymphs were reared on wheat leaves infiltrated with *P. fluorescens* EtHAn, and each aphid was weighed on a microbalance (Sartotius, GPC, Germany) after 7 days. Fifteen replicates were performed for each group. The wheat plants were replaced by new seedlings after 4 days; these seedlings received the same treatment as above. Wheat plants treated with *P. fluorescens* EtHAn carrying an empty vector were used as controls.

### RNAi application through the spray method of the transdermal dsRNA delivery system

The dsRNA specific for *Sg2204* and the homologous genes was synthesized and purified using a TranscriptAid T7 High Yield Transcription Kit (Thermo Fisher Scientific, Waltham, CA) according to the manufacturer's instructions with the primers listed in Table S4. All dsRNA sequences were labelled using the Fluorescein RNA Labeling Mix Kit (Sigma‐Aldrich, St Louis, MO). The star polycation (SPc) nanocarrier was gently mixed with dsRNA at a recommended mass ratio of 1 : 1, and 0.05% detergent (surfactant and softened water) was then added to reduce the surface tension of hydrophilic nanocomplexes (Li *et al*., 2019; Yan *et al*., 2020). The dsRNA/nanocarrier/detergent formulations with 200 ng/μL dsRNA were sprayed on the aphids and leaf surface. Aphids were then collected at 24 or 48 h after treatment with dsRNA to detect the transcript levels of target genes as described above. The primers used for RT‐qPCR are presented in Table S4.

To investigate the uptake of fluorescein labellling dsRNA in *S. graminum*, aphids were washed in distilled water for five times, and then the salivary glands and midguts of aphids were dissected in cold phosphate buffered saline (pH 7.2, HyClone, Thermo Scientific, MA) using a microscope (Olympus SZX16). The fluorescent signal in tissues was observed at 12 h after the spray of fluorescein‐labelled dsRNA/nanocarrier/detergent solution on aphids using a fluorescence microscope (Olympus BX63). Aphids sprayed with a H_2_O/nanocarrier/detergent formulation were used as a control.

To examine the effects of gene silencing on aphid performance, ten apterous adult aphids were transferred to each plant and sprayed with 200 ng/μL dsRNA. The number of surviving aphids on plants was counted after 6 days of dsRNA treatment, and ten biological replicates were performed. Five apterous adult aphids on each plant were also treated with dsRNA, and the number of nymphs produced by the aphids was recorded daily for 5 days. Fifteen replicates were conducted for each group. DsGFP was included as the control.

### Aphid feeding behaviour monitoring by electronic penetration graph (EPG)

The feeding behaviour of the adult apterous aphids on wheat plants was recorded using EPG (DC‐EPG Giga‐8). EPG monitoring was recorded continuously for 8 h in a Faraday cage in the laboratory (20 ± 1 °C, L : D = 16 : 8 photoperiod, 70% RH). Each aphid and plant were used only once. The visualization and manual labeling of the various feeding waves were carried out using Stylet+a. The characteristics of the aphid feeding waveform patterns were identified as described previously (Prado and Tjallingii, 1994). At least 10 independent biological replicates were conducted for each treatment.

### Statistical analysis

All data were analysed using SPSS Statistics 20.0 software (SPSS Inc., Chicago, IL). The differences among groups were examined using Student's *t* test and one‐way analysis of variance (ANOVA) test, followed by Duncan's multiple range test. EPG data were analysed by a Mann–Whitney *U* test. *P* values < 0.05 were considered to be statistically significant.

## Conflicts of interests

The authors declare no conflict of interest.

## Author contributions

J. L. C. and Y. Z. conceived and designed the research. Y. Z., X. B. L. and Y. S. carried out the experiment. Y. Z., F. J., Q. W. and H. L. analysed the data. Y. Z. and J. L. C. wrote the manuscript. F. F., J. L. C. and H. C. X. revised the manuscript. All authors read and approved the final manuscript.

## Supporting information


**Figure S1** Length distribution of transcripts and unigenes in transcriptome assembly for *S*. *graminum* salivary glands.
**Figure S2** Results of similarity search of unigenes against Nr database.
**Figure S3** Functional annotation of unigenes from *S*. *graminum* salivary glands using Gene Ontology (GO).
**Figure S4** Metabolic pathway analysis of by unigenes from *S*. *graminum* salivary glands using Kyoto Encyclopedia of Genes and Genomes (KEGG).
**Figure S5** RT‐qPCR results of the relative gene expression of nine candidate effectors in different tissues of *S. graminum*.

**Table S1** The quality of *S*. g*raminum* salivary glands unigene sequences and assembly.
**Table S2** Number of unigenes annotated in seven public databases.
**Table S3** Identification of watery salivary proteins of *S. graminum* using LC–MS/MS.
**Table S4** All primers used in this study.

**Data S1** All unigenes of salivary gland of *Schizaphis graminum*.
