# Supplementary material for: The salivary effector protein Sg2204 in the greenbug Schizaphis graminum suppresses wheat defence and is essential for enabling aphid feeding on host plants
Source: Plant Biotechnol J. 2022 Aug 19;20(11):2187–201. doi: 10.1111/pbi.13900 (PMC9616526; doi:10.1111/pbi.13900)
Supplement: Supplementary file 1 — Figure S1 Length distribution of transcripts and unigenes in transcriptome assembly for S. graminum salivary glands. Figure S2 Results of similarity search of unigenes against Nr database. Figure S3 Functional annotation of unigenes from S. graminum salivary glands using Gene Ontology (GO). Figure S4 Metabolic pathway analysis of by unigenes from S. graminum salivary glands using Kyoto Encyclopedia of Genes and Genomes (KEGG). Figure S5 RT‐qPCR results of the relative gene expression of nine candidate effectors in different tissues of S. graminum. [file PBI-20-2187-s003.docx]

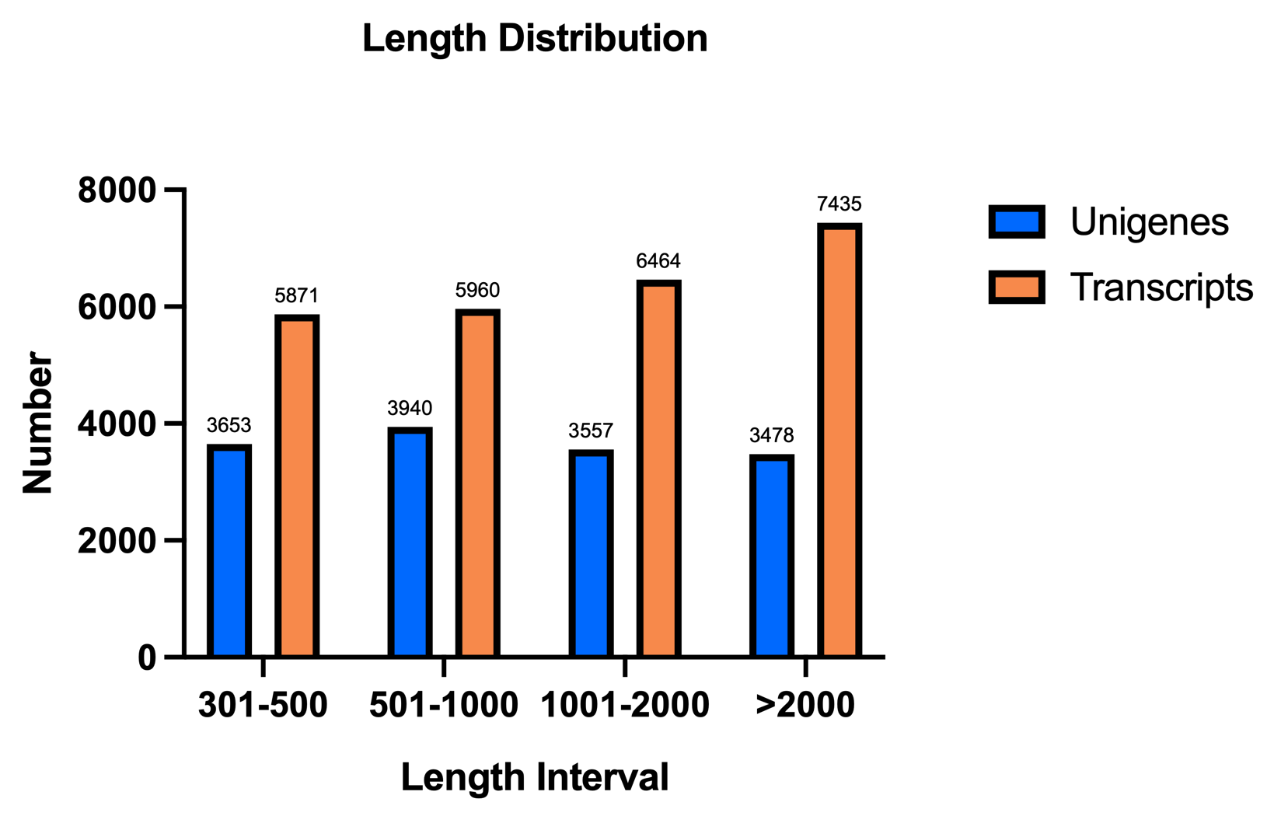


**Figure S1** Length distribution of transcripts and unigenes in transcriptome assembly for *S*. *graminum* salivary glands.


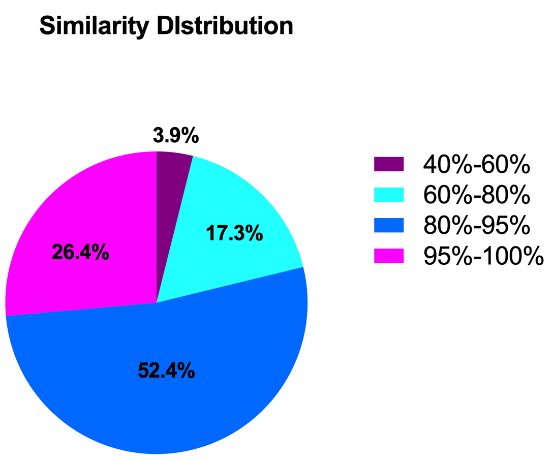

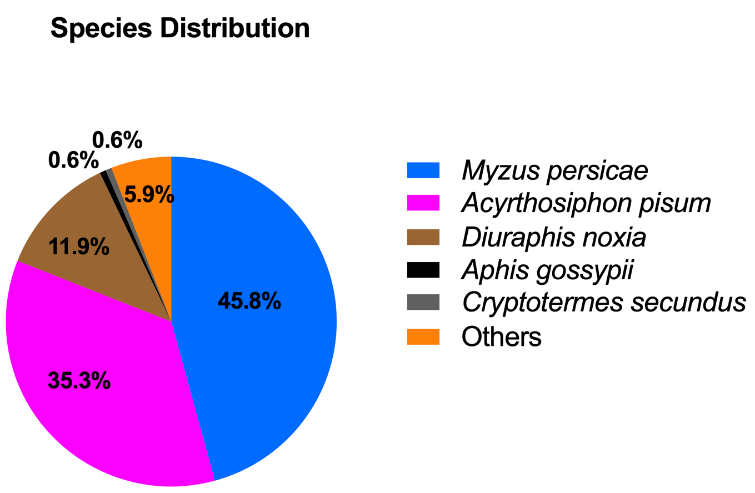

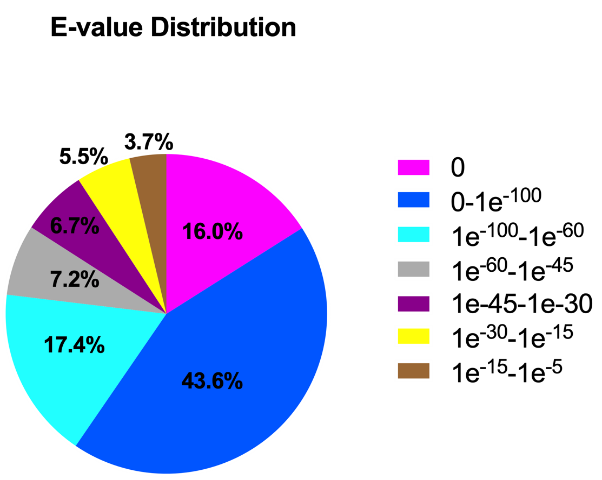


**Figure S**2 Results of similarity search of unigenes against Nr database. (A) Species distribution of the top BLAST hits for each unigene in Nr database. (B) Similarity distribution of the top BLAST hits for each unigene. (C) E-value distribution of BLAST hits for each unigene with a E-value cut off of 1.0E−5.

**
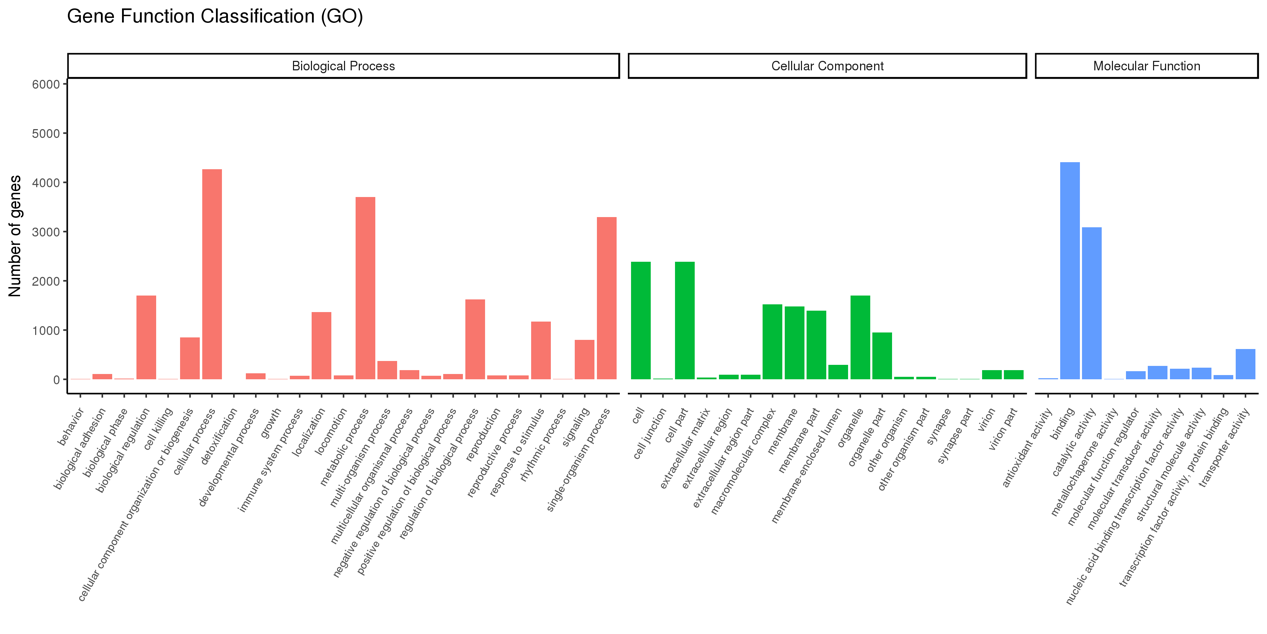
**

**Figure S3** Functional annotation of unigenes from *S*. *graminum* salivary glands using Gene Ontology (GO).

**
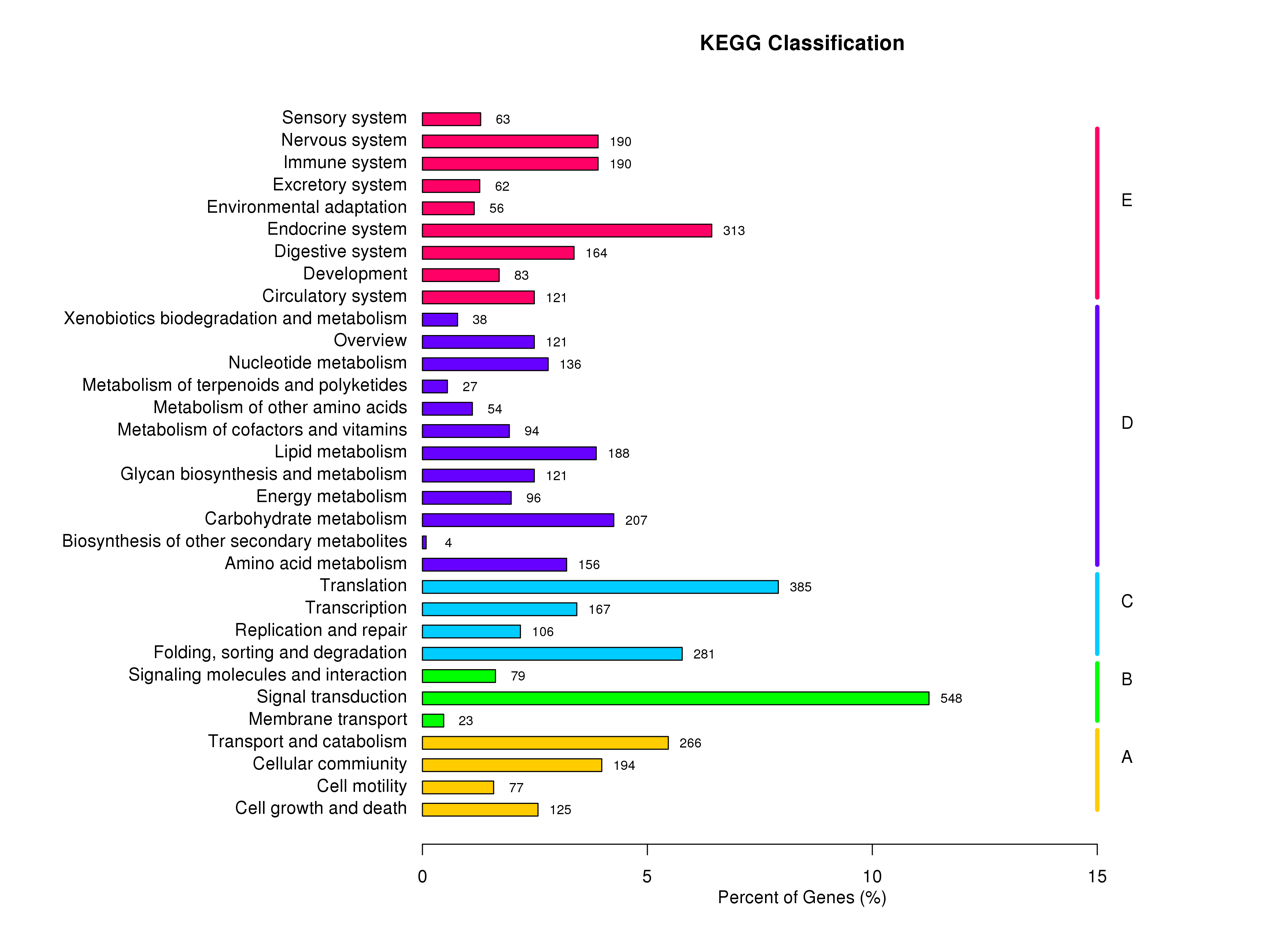
**

**Figure S4** Metabolic pathway analysis of by unigenes from *S*. *graminum* salivary glands using Kyoto Encyclopedia of Genes and Genomes (KEGG) .


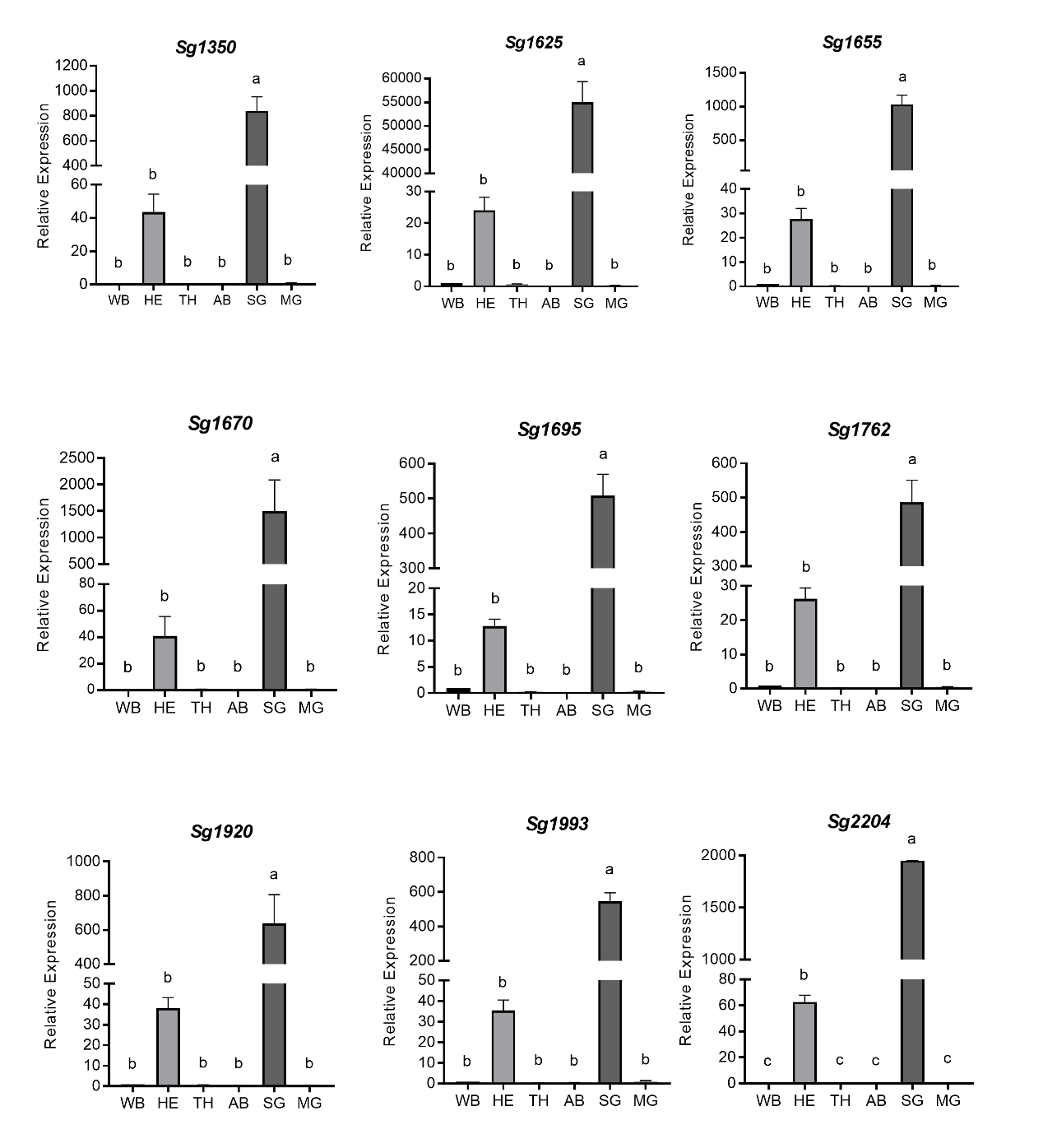


**Figure S5** RT-qPCR results of the relative gene expression of nine candidate effectors in different tissues of *S. graminum*. Abbreviation for tissues: whole body of apterous adult (WB), heads (HE), thorax (TH), abdomen (AB), salivary glands (SG), midguts (MG). β-actin and NADH were used as internal reference genes. Standard error (SE) is represented by the error bar. Different lower-case letters above each bar indicate significant differences among groups (one-way ANOVA followed by Duncan’s multiple range tests, *P*<0.05).
