# Supplementary material for: The salivary effector protein Sg2204 in the greenbug Schizaphis graminum suppresses wheat defence and is essential for enabling aphid feeding on host plants
Source: Plant Biotechnol J. 2022 Aug 19;20(11):2187–201. doi: 10.1111/pbi.13900 (PMC9616526; doi:10.1111/pbi.13900)
Supplement: Supplementary file 2 — Table S1 The quality of S. graminum salivary glands unigene sequences and assembly. Table S2 Number of unigenes annotated in seven public databases. Table S3 Identification of watery salivary proteins of S. graminum using LC–MS/MS. Table S4 All primers used in this study. [file PBI-20-2187-s002.docx]

**Table S1** The quality of *S*. g*raminum* salivary glands unigene sequences and assembly.

| **Statistics** | ***S. graminum* salivary glands** |
| --- | --- |
| Raw reads (bp) | 62276916 |
| Clean reads (bp) | 61594644 |
| Clean bases (G) | 9.24 |
| Error (%) | 0.03 |
| Q (20%) | 97.97 |
| Q (30%) | 93.67 |
| GC content (%) | 38.14 |
| Total unigenes | 14628 |

**Table S2** Number of unigenes annotated in seven public databases.

|  | **Number of Unigenes** | **Percentage (%)** |
| --- | --- | --- |
| Annotated in NR | 10157 | 69.43 |
| Annotated in NT | 9943 | 67.97 |
| Annotated in KO | 4867 | 33.27 |
| Annotated in SwissProt | 7421 | 50.73 |
| Annotated in PFAM | 7390 | 50.51 |
| Annotated in GO | 7390 | 50.51 |
| Annotated in KOG | 5148 | 35.19 |
| Annotated in all Databases | 3270 | 22.35 |
| Annotated in at least one Database | 11228 | 76.75 |
| Total Unigenes | 14628 | 100 |

**Table S3** Identification of watery salivary proteins of *S. graminum* using LC-MS/MS.

| **No.** | **Proteins** | **Unigenes** | **No. of peptides** | **No. of Unique peptides** | **Unique sequence coverage [%]** | **Mol. weight [kDa]** | **Protein score** |
| --- | --- | --- | --- | --- | --- | --- | --- |
| **1** | sheath protein | Cluster-5206.1209 | 2 | 2 | 43.1 | 5.9224 | 224.68 |
| **2** | Me10-like (LOC111033418) | Cluster-5206.1661 | 4 | 4 | 25.9 | 13.435 | 160.99 |
| **3** | DNA-directed RNA polymerase | Cluster-5206.1960 | 10 | 10 | 61.8 | 14.518 | 170.37 |
| **4** | uncharacterized protein LOC111030505 | Cluster-5206.1655 | 2 | 2 | 24.4 | 14.898 | 33.098 |
| **5** | uncharacterized protein LOC100570017 | Cluster-5206.2204 | 3 | 3 | 29.3 | 16.708 | 20.852 |
| **6** | yellow-like isoform X2 | Cluster-6239.0 | 5 | 5 | 39.5 | 17.161 | 74.437 |
| **7** | uncharacterized protein LOC100168922 | Cluster-5206.1350 | 5 | 5 | 36.4 | 18.244 | 40.901 |
| **8** | uncharacterized protein LOC100573979 | Cluster-5206.1821 | 2 | 2 | 11 | 18.736 | 12.726 |
| **9** | C002-like | Cluster-5206.1621 | 3 | 3 | 25.3 | 19.44 | 26.424 |
| **10** | uncharacterized protein LOC100569633 | Cluster-5206.1443 | 4 | 2 | 13.1 | 19.633 | 62.031 |
| **11** | uncharacterized protein LOC107168866 | Cluster-5206.1491 | 3 | 3 | 17.8 | 20.16 | 29.665 |
| **12** | uncharacterized protein | Cluster-5206.1920 | 8 | 8 | 41.6 | 20.318 | 80.357 |
| **13** | uncharacterized protein LOC100166545 | Cluster-5206.1625 | 5 | 5 | 31.5 | 22.682 | 64.101 |
| **14** | brevican core protein | Cluster-5206.1708 | 6 | 6 | 31.2 | 23.345 | 199.52 |
| **15** | uncharacterized protein LOC111035063 | Cluster-5206.1695 | 7 | 7 | 43.8 | 23.868 | 55.116 |
| **16** | beta-actin | Cluster-7785.0 | 4 | 1 | 13.7 | 24.16 | 55.076 |
| **17** | vascular endothelial growth factor A-A-like | Cluster-8100.0 | 2 | 2 | 4.7 | 24.44 | 12.899 |
| **18** | sheath protein | Cluster-5206.1623 | 22 | 22 | 91.6 | 24.561 | 323.31 |
| **19** | uncharacterized protein LOC100569669 | Cluster-5206.1913 | 3 | 3 | 13.3 | 25.05 | 60.625 |
| **20** | BerH2-scFv-hpRNase precursor | Cluster-3951.0 | 5 | 5 | 30.1 | 25.446 | 72.509 |
| **21** | glutathione peroxidase (LOC100161198) | Cluster-5206.1696 | 8 | 8 | 38 | 26.48 | 323.31 |
| **22** | uncharacterized protein LOC107168676 | Cluster-5206.1993 | 2 | 2 | 9 | 27.163 | 11.981 |
| **23** | histone H2A | Cluster-5206.2654 | 2 | 2 | 6.5 | 29.03 | 13.829 |
| **24** | uncharacterized protein LOC111026940 | Cluster-5206.1762 | 5 | 5 | 15.4 | 29.921 | 323.31 |
| **25** | sheath protein  (LOC100169243) | Cluster-5206.1675 | 24 | 23 | 58.1 | 29.951 | 323.31 |
| **26** | yellow protein | Cluster-6182.0 | 3 | 3 | 13.8 | 30.026 | 25.887 |
| **27** | uncharacterized protein ACYPI000490 | Cluster-5206.1670 | 12 | 12 | 52.9 | 30.197 | 223.26 |
| **28** | uncharacterized protein LOC100569633 | Cluster-5206.1860 | 7 | 5 | 21.2 | 31.194 | 294.11 |
| **29** | uncharacterized protein LOC100169243 | Cluster-5206.1664 | 7 | 6 | 33.7 | 31.525 | 132.85 |
| **30** | uncharacterized protein LOC100573887 | Cluster-5206.1812 | 6 | 6 | 28.7 | 32.78 | 62.236 |
| **31** | uncharacterized protein LOC111031336 | Cluster-5206.1692 | 8 | 8 | 37.5 | 32.949 | 143.77 |
| **32** | peroxidase-like | Cluster-5206.1879 | 3 | 3 | 12.2 | 34.239 | 38.251 |
| **33** | lipase, partial | Cluster-5206.1615 | 3 | 3 | 8.1 | 34.389 | 30.643 |
| **34** | prostatic spermine-binding protein | Cluster-5206.1721 | 3 | 3 | 11.2 | 35.201 | 19.865 |
| **35** | alpha-galactosidase | Cluster-5206.611 | 2 | 2 | 8 | 36.798 | 12.662 |
| **36** | bromodomain adjacent to zinc finger domain protein 2B | Cluster-5206.1781 | 2 | 2 | 8.9 | 37.489 | 15.766 |
| **37** | cathepsin B | Cluster-5206.1714 | 3 | 3 | 14.5 | 37.805 | 56.438 |
| **38** | PE-PGRS family protein | Cluster-5206.1519 | 2 | 2 | 9.9 | 38.277 | 29.087 |
| **39** | AP2/ERF domain-containing protein PFD0985w | Cluster-5206.2140 | 6 | 6 | 18.1 | 39.454 | 60.162 |
| **40** | uncharacterized protein LOC100572887 | Cluster-5206.1156 | 4 | 4 | 11.6 | 39.74 | 22.973 |
| **41** | lipase-like | Cluster-5206.509 | 4 | 4 | 10.8 | 40.803 | 26.408 |
| **42** | peroxidase-like | Cluster-5206.1879 | 10 | 10 | 38.8 | 41.167 | 247.6 |
| **43** | uncharacterized protein LOC103308649 | Cluster-5206.1639 | 3 | 3 | 7.1 | 41.57 | 29.229 |
| **44** | actin | Cluster-5206.2172 | 11 | 8 | 21 | 41.821 | 122.81 |
| **45** | uncharacterized protein LOC100162171 | Cluster-5206.1698 | 2 | 2 | 7.5 | 41.916 | 11.446 |
| **46** | uncharacterized protein LOC100163848 | Cluster-5206.1790 | 8 | 8 | 27.5 | 41.959 | 153.46 |
| **47** | yellow protein | Cluster-5206.1197 | 2 | 2 | 5.1 | 42.615 | 12.877 |
| **48** | carbonic anhydrase 2 | Cluster-5206.2363 | 5 | 5 | 12.8 | 43.33 | 42.557 |
| **49** | ACYPI008246 | Cluster-5206.2992 | 3 | 3 | 16.7 | 43.347 | 43.863 |
| **50** | lipase (LOC107882302) | Cluster-5206.2178 | 2 | 2 | 9.7 | 43.757 | 15.044 |
| **51** | uncharacterized protein LOC107882155 | Cluster-5206.1686 | 5 | 5 | 22.1 | 44.959 | 83.932 |
| **52** | uncharacterized protein LOC100158692 | Cluster-5206.1631 | 7 | 7 | 16.9 | 45.096 | 234.18 |
| **53** | uncharacterized protein LOC111033342 | Cluster-5206.1546 | 3 | 3 | 11.5 | 46.061 | 27.028 |
| **54** | uncharacterized protein LOC100572954 | Cluster-5206.1635 | 9 | 9 | 28.9 | 46.559 | 109.4 |
| **55** | alpha-N-acetylgalactosaminidase | Cluster-7055.0 | 11 | 10 | 28.3 | 49.792 | 86.6 |
| **56** | carboxypeptidase E-like | Cluster-5206.1537 | 4 | 4 | 11.5 | 54.583 | 35.949 |
| **57** | uncharacterized protein LOC100162791 | Cluster-5206.1761 | 21 | 21 | 52 | 62.825 | 295.23 |
| **58** | uncharacterized protein LOC111034237 | Cluster-5206.1765 | 6 | 6 | 12.8 | 65.017 | 112.51 |
| **59** | uncharacterized protein LOC111027222 | Cluster-5206.1633 | 15 | 15 | 20 | 68.879 | 323.31 |
| **60** | chitooligosaccharidolytic beta-N-acetylglucosaminidase | Cluster-5206.2076 | 8 | 8 | 21.6 | 69.013 | 70.655 |
| **61** | glucose dehydrogenase | Cluster-5206.1989 | 45 | 34 | 46.2 | 69.592 | 323.31 |
| **62** | soluble trehalase | Cluster-5206.1646 | 30 | 30 | 50.7 | 71.639 | 323.31 |
| **63** | uncharacterized protein LOC111026863 | Cluster-5206.1595 | 11 | 11 | 20.7 | 72.442 | 177.98 |
| **64** | serine/threonine-protein kinase | Cluster-5206.1571 | 2 | 2 | 3.5 | 73.704 | 32.786 |
| **65** | peroxidase | Cluster-5206.1531 | 18 | 18 | 35.3 | 73.99 | 237.49 |
| **66** | uncharacterized protein LOC100571147 | Cluster-5206.1673 | 12 | 12 | 25.5 | 75.154 | 247.72 |
| **67** | glucose dehydrogenase | Cluster-5206.1535 | 61 | 53 | 58.6 | 80.287 | 323.31 |
| **68** | neuroendocrine convertase 1 | Cluster-5206.1963 | 4 | 4 | 5.2 | 83.192 | 29.315 |
| **69** | structural polyprotein | Cluster-5206.1871 | 20 | 20 | 37.2 | 87.228 | 310.96 |
| **70** | cyclin-dependent serine/threonine-protein kinase | Cluster-5206.1712 | 35 | 35 | 48.9 | 103.38 | 323.31 |
| **71** | lysosomal alpha-mannosidase | Cluster-5206.2248 | 13 | 13 | 14.3 | 115.06 | 98.036 |
| **72** | extracellular matrix-binding protein | Cluster-5206.1506 | 22 | 21 | 20.8 | 120.41 | 297.69 |
| **73** | glucose-methanol-choline oxidoreductase (LOC111035667) () | Cluster-5206.1579 | 105 | 93 | 71.4 | 123.55 | 323.31 |
| **74** | uncharacterized protein PF11_0213 | Cluster-5206.1532 | 22 | 22 | 20.5 | 177.26 | 323.31 |
| **75** | uncharacterized protein LOC100573887 | Cluster-5206.190 | 43 | 43 | 31.8 | 215.03 | 323.31 |
| **76** | apolipophorins | Cluster-5206.1559 | 146 | 139 | 45.9 | 321.23 | 323.31 |

**Table S4** All primers used in this study.

| **Primer name** | **Primer sequence (5’-3’)** | **Purpose** |
| --- | --- | --- |
| Sg1350-qPCR | F: ATTGCACAATCAGACGGTGA  R: AGATGCTTCCTTGGCGAAAT | qPCR for aphids |
| Sg1625-qPCR | F: TGCAATCGTTTCGCTAACAG  R: GTTGGCACCTTTCATCACCT |
| Sg1655-qPCR | F: CAAAGCTGACGACGTTCAAA  R: TCATTGGATCATTTCCGACA |
| Sg1670-qPCR | F: GCAGGGATTCAAAGAAGTGG  R: ACATACCGCCCATGTTTAGC |
| Sg1695-qPCR | F: TTTTCACCGTTGCCAATACA  R: AATTCGTTGGCCATTTCATC |
| Sg1762-qPCR | F: CGGTGGCTACAAACGATACA  R: TCATCCTCGTCCAAATTTCC |
| Sg1920-qPCR | F: CGACGGAAAATGTGGATTCT  R: GGTCCAGGAAGTCGTCGTAA |
| Sg1993-qPCR | F: GGGGTGTCGAACAAGGTGTA  R: CGATCCCGGTGTTGAGTATT |
| Sg2204-qPCR | F: ATTTGCCATTCGAGTTCAGC  R: CTGGCAGGAATGGGAGAATA |
| NADH dehydrogenase | F: CGAGGAGAACATGCTCTTAGAC  R: GATAGCTTGGGCTGGACATATAG |
| β-actin | F: CGGTTCAAAAACCCAAACCAG  R: TGGTGATGATTCCCGTGTTC |
| pCAMBIA1300-Sg2240 | F: CGAGCTC ATGTCAAGAATGCCAGATTCA  R: GGGGTACC AACATACTTGCTAAAGTTT | Subcellular localization |
| pGR107-INF1 | F: CCATCGATATGAACTTTCGTGCTCTGTTCG  R: TCCCCCGGGTCATAGCGACGCACACGTAGA | Transient overexpression |
| pGR107-BAX | F: TCCCCCGGGATGGACGGGTCCGGGGA  R: ACGCGTCGACTCAGCCCATCTTCTTCCAGAT |
| pGR107-Sg2204 | F: TCCCCCGGGATGTCAAGAATGCCAGATTCA  R: ACGCGTCGACTTAAACATACTTGCTAAAGTTT |
| pGR107-GFP | F: TCCCCCGGGATGGTGAGCAAGGGCGA  R: ACGCGTCGACTTACTTGTACAGCTCGTCCATG |
| pEDV6-Sg2204 | F: GGGGACAAGTTTGTACAAAAAAGCAGGCTTC  TCAAGAATGCCAGATTCA  R: GGGGACCACTTTGTACAAGAAAGCTGGGTC  TTAAACATACTTGCTAAAGTTT | Deliverin1g by T3SS |
| pEDV6-Sa2204 | F: GGGGACAAGTTTGTACAAAAAAGCAGGCTTC  CTTACTTCGACCGCCGCAA  R: GGGGACCACTTTGTACAAGAAAGCTGGGTC  TCAAATGTATTCAATTCTCA |
| pEDV6-Rm2204 | F: GGGGACAAGTTTGTACAAAAAAGCAGGCTTC GCAAGAATGCCAGATTCAGAT  R: GGGGACCACTTTGTACAAGAAAGCTGGGTC TTAAATGTATTCAATTCTC |
| pEDV6-Mp2204 | F: GGGGACAAGTTTGTACAAAAAAGCAGGCTTC GGAAAAGTGCCATCTTCAGAT  R: GGGGACCACTTTGTACAAGAAAGCTGGGTC TCAAATGTATTCAATTCTCAT |
| pEDV6-Ap2204 | F: GGGGACAAGTTTGTACAAAAAAGCAGGCTTC GCAAGAATGCCCGATTCAG  R: GGGGACCACTTTGTACAAGAAAGCTGGGTC TCAAACGTATTCAATTCTCA |
| FAD | F: TCCCATTCCACCTACTGC  R: GGACTCACCAATCCGAGA | qPCR for wheat |
| LOX | F: GACCAGCGAAACAACAACC  R: GCATACAATAGCGGGAACAC |
| PAL | F: CCACCCTGGACAGATTGAA  R: ATGAGCGGGTTGTCGTTG |
| PR1 | F: ATAACCTCGGCGTCTTCAT  R: TACTCGCTCGGTCCCTCT |
| β-actin | F: GGAAAATCAGTCTCGGTTCAG  R: TCATACAGCAGGCAAGCAC |
| Sg2204-T7 | F: TAATACGACTCACTATAGGG  ATGACGACGATTACATC  R:TAATACGACTCACTATAGGG  TTAAACATACTTGCTAAAGT | RNAi |
| GFP-T7 | F: TAATACGACTCACTATAGGG  TACGGCGTGCAGTGCT  R: TAATACGACTCACTATAGGG  TGATCGCGCTTCTCG |
